# Human induced pluripotent stem cell‐derived versus adult cardiomyocytes: an *in silico* electrophysiological study on effects of ionic current block

**DOI:** 10.1111/bph.13282

**Published:** 2015-10-15

**Authors:** M Paci, J Hyttinen, B Rodriguez, S Severi

**Affiliations:** ^1^ Department of Electronics and Communications EngineeringTampere University of Technology, BioMediTechTampereFinland; ^2^ Department of Computer ScienceUniversity of OxfordOxfordUK; ^3^ Department of Electrical, Electronic and Information Engineering ‘Guglielmo Marconi’University of BolognaCesena (FC)Italy

## Abstract

**Background and Purpose:**

Two new technologies are likely to revolutionize cardiac safety and drug development: *in vitro* experiments on human‐induced pluripotent stem cell‐derived cardiomyocytes (hiPSC‐CMs) and *in silico* human adult ventricular cardiomyocyte (hAdultV‐CM) models. Their combination was recently proposed as a potential replacement for the present hERG‐based QT study for pharmacological safety assessments. Here, we systematically compared *in silico* the effects of selective ionic current block on hiPSC‐CM and hAdultV‐CM action potentials (APs), to identify similarities/differences and to illustrate the potential of computational models as supportive tools for evaluating new *in vitro* technologies.

**Experimental Approach:**

*In silico* AP models of ventricular‐like and atrial‐like hiPSC‐CMs and hAdultV‐CM were used to simulate the main effects of four degrees of block of the main cardiac transmembrane currents.

**Key Results:**

Qualitatively, hiPSC‐CM and hAdultV‐CM APs showed similar responses to current block, consistent with results from experiments. However, quantitatively, hiPSC‐CMs were more sensitive to block of (i) L‐type Ca^2+^ currents due to the overexpression of the Na^+^/Ca^2+^ exchanger (leading to shorter APs) and (ii) the inward rectifier K^+^ current due to reduced repolarization reserve (inducing diastolic potential depolarization and repolarization failure).

**Conclusions and Implications:**

*In silico* hiPSC‐CMs and hAdultV‐CMs exhibit a similar response to selective current blocks. However, overall hiPSC‐CMs show greater sensitivity to block, which may facilitate *in vitro* identification of drug‐induced effects. Extrapolation of drug effects from hiPSC‐CM to hAdultV‐CM and pro‐arrhythmic risk assessment can be facilitated by *in silico* predictions using biophysically‐based computational models.

**Abbreviations**
AL: atrial‐like
APD: AP duration
APD_ratio_: AP shape factor
EAD: early after depolarizations
hAdultV‐CM: human adult ventricular cardiomyocyte
hiPSC: human‐induced pluripotent stem cell
hiPSC‐CM: hiPSC‐derived cardiomyocyte
MDP: maximum diastolic potential
ORd: O'Hara–Rudy model of human adult ventricular cell
Paci2013: Paci model of hiPSC‐CM
Rate: rate of spontaneous beating
VL: ventricular‐like
V_Max_: maximum upstroke velocity

## Tables of Links

**Table:** 

TARGETS
**GPCRs** *^b^*
β‐adrenoceptors
**Transporters** *^b^*
Na+/Ca2+ exchanger

LIGANDS		
Chromanol 293B	Ivabradine	Nisoldipine
Dofetilide	Mexiletine	ORM10103
E4031	Nickel	TTX
HMR‐1556	Nifedipine	Zatebradine

These Tables list key protein targets and ligands in this article which are hyperlinked to corresponding entries in http://www.guidetopharmacology.org, the common portal for data from the IUPHAR/BPS Guide to PHARMACOLOGY (Pawson *et al*., 2014) and are permanently archived in the Concise Guide to PHARMACOLOGY 2013/14 (^*a,b*^Alexander *et al*., 2013a, 2013b).

## Introduction

Preclinical evaluation of the safety and efficacy of compounds before doing costly clinical trials is a major concern for pharmaceutical and cosmetic industry, regulators and society as a whole. Of major importance is the assessment of the effects of all new compounds on the heart, specifically for the identification of possible cardiotoxic side effects such as pro‐arrhythmia (Heist and Ruskin, 2010; Kannankeril, 2010; Sauer and Newton‐Cheh, 2012; Shah, 2013). Currently, pro‐arrhythmic risk is initially assessed preclinically using ion channel assays (especially hERG assays) and evaluating the prolongation of the cardiac action potential (AP) duration, which is manifested in the ECG as the QT interval (Leishman *et al*., 2012; Hamdam *et al*., 2013). *In vivo* and *ex vivo* experiments using animals (rats, rabbits, dogs and non‐human primates) are also used, in spite of two major limitations (Rodriguez *et al*., 2010): (i) impaired translation from animal experiments to humans due to inter‐species differences in the underlying physiology, and (ii) a lack of heterogeneity and diseased specimens in the samples considered in the experiments compared with the huge variability in the human population.

Two novel technologies have been proposed as a promising way to overcome the limitations of the existing methodologies used for preclinical safety evaluation of pharmaceutical compounds: *in vitro* experiments using human‐induced pluripotent stem cell‐derived cardiomyocytes (hiPSC‐CMs) and *in silico* simulations using human adult ventricular cardiomyocyte (hAdultV‐CM) models. The potential of both techniques, and especially their combination, has been acknowledged by the pharmaceutical industry, and also by the recent announcement by the US Food and Drug administration of a new paradigm for the evaluation of new molecular entities: ‘the comprehensive *in vitro* pro‐arrhythmia assay’ (Sager *et al*., 2014). The proposed scheme would include the following: (i) screening of drug action on multiple human cardiac currents (rather than just hERG) in heterologous expression systems; (ii) integration of ion channel/drug interaction data in *in silico* models of human ventricular electrophysiology to predict and evaluate changes in the human action potential; and (iii) *in vitro* evaluation of compound effects in a myocyte assay such as hiPSC‐CMs and comparison to the *in silico* results. A critical point not assessed so far is how electrophysiological differences between hiPSC‐CM and hAdultV‐CM may result in different drug responses. Identifying these differences is critical to ensure the interpretation of combined hiPSC‐CM and hAdultV‐CM *in vitro*/*in silico* assays.

In this study, we have conducted the first systematic *in silico* evaluation of the effects of selective ionic current block on the APs of ventricular‐like (VL) and atrial‐like (AL) hiPSC‐CMs and hAdultV‐CMs. Our ultimate aim was to inform the potential of *in vitro* and *in silico* hiPSC‐CMs technologies for preclinical drug evaluation. Our focus on selective ionic current block facilitates the systematic comparison of the different models and allows the quantification of the sensitivity of the AP to the inhibition of specific ionic currents. In our investigations we considered the most recent computational human models, which integrate existing biophysically detailed knowledge and datasets on the membrane kinetics of both hiPSC‐CMs and hAdultV‐CMs. We identified similarities and differences in the response of both types of cardiomyocytes to ionic current block and discuss them with regard to the underlying ionic mechanisms, in order to facilitate the interpretation of future experiments.

## Methods

### In silico models

Spontaneous and stimulated hiPSC‐CM APs were simulated using the Paci2013 model (Paci *et al*., 2013a), based on the comprehensive experimental dataset reported by Ma *et al*. (2011), obtained on isolated iCell hiPSC‐CMs (Cellular Dynamics International, Madison, WI, USA). The Paci2013 model allows the simulation of both VL and AL hiPSC‐CMs AP and includes formulations for the dynamics of ionic concentrations in the cytoplasm and sarcoplasmic reticulum, and for the kinetics of the main ionic currents and pumps, as described in the original paper. The VL and AL models were developed taking into account the differences between the main currents in ventricular and atrial phenotypes. This was performed by using scaling factors based on available data on known differences between ventricular and atrial cells. Importantly, the VL and AL models were independently validated against the morphological features of the APs for each cardiac phenotype reported previously (Ma *et al*., 2011) (Table 1). Slight improvements in the Paci2013 model were included, as described in the Supporting Information.

**Table 1:** Comparison between the morphological biomarkers in spontaneous and stimulated VL hiPSC‐CM and AL hiPSC‐CM APs and Ca^2+^ transients

	EXP Non‐stimulated (Ma *et al*., 2011)		SIM				
			Non‐stimulated		Stimulated		
	hiPSC‐CM		hiPSC‐CM		hiPSC‐CM		hAdultV‐CM
	AL	VL	AL	VL	AL	VL	
MDP (mV)	−73.5 ± 1.5	−75.6 ± 1.2	−72.2	−77.4	−71.3	−76.2	−88.0
V_Max_ (V s^−1^)	26.2 ± 3.9	27.8 ± 4.8	24.9	26.3	33.9	47.4	259
APA (mV)	100 ± 2	104 ± 1	99.1	105	100	115	128
Peak (mV)	26.7 ± 1.4	28.3 ± 1.0	26.9	27.5	28.8	38.5	40.0
APD_30_ (ms)	123 ± 10	180 ± 11	137	212	167	258	166
APD_50_ (ms)	–	–	186	307	222	367	208
APD_70_ (ms)	–	–	229	358	267	418	240
APD_90_ (ms)	286 ± 21	415 ± 22	301	399	357	469	268
APD_ratio_	1.1 ± 0.1 (<1.5)	2.5 ± 0.2 (>1.5)	1.09	3.16	1.09	3.41	1.60
Rate (beats min^−1^)	50.0 ± 10.0	35.3 ± 2.2	55.2	37.3	–	–	–
Diastolic Ca^2+^ (nm)	–	–	38	16	40	11	86
Systolic Ca^2+^ (nm)	–	–	296	281	241	150	368
Amplitude (nm)	–	–	258	265	201	140	282

EXP, experimental; SIM, simulated; APA, AP amplitude; Peak, peak voltage; Rate, rate of the spontaneous electrical activity; VL, ventricular‐like; AL, atrial‐like; hiPSC‐CM, human‐induced pluripotent stem cell‐derived cardiomyocyte; APs, action potentials; APD, action potential duration.

*In silico* simulations were also conducted using the hAdultV‐CM O'Hara–Rudy (ORd) endocardial model (O'Hara *et al*., 2011). The ORd model includes a similar level of biophysical detail as the Paci2013 model.

### AP assessment

Action potentials were evaluated at steady‐state, after 900 s of simulation. The following morphological AP biomarkers were quantified: AP amplitude, AP duration at several repolarization percentages (APD_30_, APD_50_, APD_70_, and APD_90_), maximum diastolic potential (MDP), maximum upstroke velocity (V_Max_), peak voltage and spontaneous beating rate. The shape factor

$$APD_{\text{ratio}}=\frac{APD_{30}-APD_{40}}{APD_{70}-APD_{80}}$$

discriminates between VL and AL hiPSC‐CM APs: as in Ma *et al*., (2011), an AP is considered VL if *APD_ratio_* > 1.5 and AL if *APD_ratio_* < 1.5. Details about the stimulation protocols are reported in the Supporting Information.

For each biomarker, the % variation induced by blocking each ionic current was computed (Tables 2, S1, S2 and S3). Then, considering only one model at a time, we normalized the variations in each specific biomarker with respect to the largest one (the most positive one for positive variations or the most negative one for negative variations) within all ionic current blocks (Figures 5, 6 and S2). To compare VL hiPSC‐CM and hAdultV‐CM, we repeated the previous procedure considering the % variations for both models (Figure 7).

**Table 2:** Comparison between the % changes caused by each ionic current block in the AP biomarkers quantified by the VL hiPSC‐CM and the hAdultV‐CM AP models

		Variation (%)								
		MDP	V_Max_	APA	Peak	APD_30_	APD_50_	APD_70_	APD_90_	APD_ratio_
*I_Na_*	VL hiPSC‐CM	1	−53	−7	−23	14	3	0	1	−20
	hAdultV‐CM	0	−69	−3	−8	3	2	2	1	0
*I_CaL_*	VL hiPSC‐CM	0	5	0	2	−82	−73	−68	−66	−35
	hAdultV‐CM	0	3	−2	−5	−24	−21	−17	−16	−5
*I_Kr_*Ad	VL hiPSC‐CM	0	−35	−6	−17	63	64	59	56	38
	hAdultV‐CM	0	0	0	1	45	56	67	66	2
*I_Kr_*SS	VL hiPSC‐CM^a^	−2	−48	−2	−2	96	86	79	69	58
	hAdultV‐CM^a^	0	1	0	1	32	39	45	44	16
*I_Ks_*	VL hiPSC‐CM	0	−1	0	−1	3	2	2	2	4
	hAdultV‐CM	0	0	0	0	5	4	4	4	−5
*I_K1_*	VL hiPSC‐CM	−16	−65	−14	−12	30	22	34	50	−73
	hAdultV‐CM	0	2	1	1	0	0	1	7	−28
*I_f_*	VL hiPSC‐CM	1	21	4	9	1	3	3	4	6
	hAdultV‐CM	–	–	–	–	–	–	–	–	–
*I_NaCa_*	VL hiPSC‐CM	−1	−1	2	6	11	1	−3	−8	24
	hAdultV‐CM	0	1	0	1	−10	−11	−10	−9	−18
*I_to_*	VL hiPSC‐CM	0	−2	1	2	2	2	2	2	7
	hAdultV‐CM	0	0	2	5	−2	−1	0	0	17

The 2 × IC_50_ concentration was considered, except where specified. The % variations with respect to the control values of each biomarker are reported. Two rows are reported for *I_Kr_*: Ad contains biomarkers 7 s after administration of the blocker, while SS refers to the steady‐state.

a IC_50_ concentration is considered.

EXP, experimental; SIM, simulated; APA, AP amplitude; Peak, peak voltage; Rate, rate of the spontaneous electrical activity; VL, ventricular‐like; AL, atrial‐like; hiPSC‐CM, human‐induced pluripotent stem cell‐derived cardiomyocyte; APs, action potentials; APD, action potential duration.

**Table 3:** Comparison between four datasets to show the variability inherent with VL hiPSC‐CM measurements

	Ma2011	Moretti2010	Lahti2011	Ma2013
Rate (beats min^‐1^)	35 ± 2	68 ± 3	72 ± 6	69 ± 11
MDP (mV)	−76 ± 1	−64 ± 2	−63 ± 1	−61 ± 1
APD_90_ (ms)	415 ± 22	381 ± 35	314 ± 18	434 ± 31

VL, ventricular‐like; hiPSC‐CM, human‐induced pluripotent stem cell‐derived cardiomyocyte; APD, action potential duration; MDP, maximum diastolic potential.

Ma2011 (Ma *et al*., 2011) includes data from control hiPSC‐CMs, used to record currents (*I_Na_*, *I_CaL_*, *I_f_*, *I_to_*, *I_Kr_*, *I_Ks_* and *I_K1_*), spontaneous APs and response to selective blocks of *I_Na_*, *I_CaL_*, *I_Kr_* and *I_Ks_*. Moretti2010 (Moretti *et al*., 2010) includes AP (spontaneous and stimulated) and current data (*I_Ks_* and *I_Kr_*) from control and patient cells to study the effects of LQT1. Lahti2011 (Lahti *et al*., 2012) shows *I_Kr_* and the spontaneous APs from control and patient hiPSC‐CMs for LQT2 assessment. Ma2013 (Ma *et al*., 2013) focuses on control and patient hiPSC‐CMs to characterize the effects of LQT3, analysing APs and the fast and late *I_Na_*.

### In silico simulation of ionic current block

Specific block of *I_Na_*, *I_CaL_*, *I_f_*, *I_K1_*, *I_Kr_*, *I_Ks_*, *I_to_* and *I_NaCa_* was assessed on stimulated APs. As in previous studies (Brennan *et al*., 2009; Davies *et al*., 2012; Zemzemi *et al*., 2013), drug block was simulated using a simple pore block model affecting the relevant maximum conductances based on a dose–response curve for each of the ionic currents, *I*, as follows:

$$\frac{I\left[D\right]}{I}=\frac{1}{1+\frac{\left[D\right]}{IC_{50}}}$$

where [*D*] represents the current blocker concentration, IC_50_ is the drug concentration leading to 50% of block and the Hill coefficient is assumed to be one. This particular model aids in the interpretation of our results for selective drugs. For each current, four different degrees of block were tested, 0.1 × IC_50_, IC_50_, 2 × IC_50_ and full block, corresponding to current block levels of 9, 50, 67 and 100%. From steady‐state conditions, the maximum conductance of each current was reduced according to the specific concentration of the blocker. Simulations were then run for an additional 300 s, after which the APs were assessed and the aforementioned biomarkers were quantified. In the case of *I_Kr_* block, APs were assessed after 7 s from block, because for longer simulations, repolarization failure was observed in some cases (at 2 × IC_50_ in AL hiPSC‐CM and at full block in VL and AL hiPSC‐CM and hAdultV‐CM).

### Numerical simulations

The Paci2013 model was implemented in MATLAB Simulink (The MathWorks, Inc., Natick, MA, USA), whereas the ORd model was implemented in MATLAB. Both models were solved using ode15s.

## Results

### Spontaneous and stimulated control hiPSC‐CM APs

**Figure 1:**
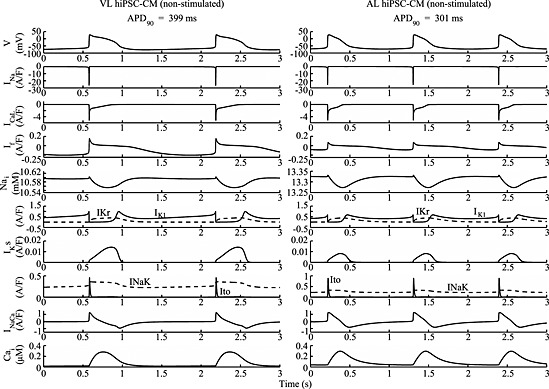
Simulated spontaneous VL (left) and AL (right) hiPSC‐CM action potentials, ionic currents and concentrations. No external stimulus was used (*I_stim_* = 0).

**Figure 2:**
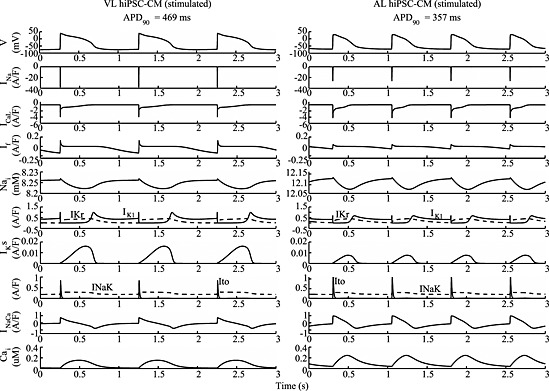
Simulated VL (left) and AL (right) hiPSC‐CM action potentials, ionic currents and concentrations under stimulation at 60  (VL) and 80 beats min^‐1^ (AL).

The time course of the simulated VL and AL hiPSC‐CM APs and the underlying ionic currents is illustrated in Figures 1 and 2 at steady‐state, for spontaneous and stimulated APs, respectively. Table 1 presents the quantitative comparison between the biomarkers obtained from experimental spontaneous APs as reported in Ma *et al*., (2011) and those quantified from the *in silico* simulations. Both models yielded values for the biomarkers consistent with experimentally‐reported ranges. Moreover, the main differences between VL and AL APs were successfully simulated, including an *APD_ratio_* of 3.16 and 1.09 and the higher spontaneous beating rate of AL compared with VL hiPSC‐CMs, as reported by Moretti *et al*., (2010), Ma *et al*., (2011) and Lahti *et al*., (2012).

The last three columns of Table 1 provide a comparison between the biomarkers from stimulated hiPSC‐CM and hAdultV‐CM APs simulations. Simulated hiPSC‐CM exhibited depolarized MDP values (by ~12 mV), decreased AP amplitude and longer APD than hAdultV‐CM, with values consistent with the experimental data reported by Li *et al*. (1998) and Moretti *et al*. (2010).

### Effects of ionic current block on hiPSC‐CM and hAdultV‐CM AP

**Figure 3:**
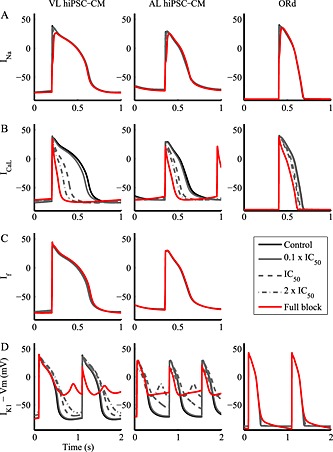
Comparison of the steady‐state effects on the action potential of ionic current block in the VL (left) and AL (centre) hiPSC‐CM and hAdultV‐CM (right) models. The four blocker concentrations considered were as follows: 0.1 × IC_50_ = 9%, IC_50_ = 50%, 2 × IC_50_ = 67% and full block. *I_f_* block was not simulated for hAdultV‐CM model because it does not contain this current. VL and AL hiPSC‐CM I_K1_ full block trace refers to the first APs after the block.

**Figure 4:**
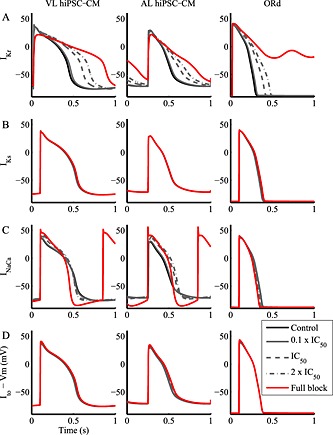
Comparison of the steady‐state effects on the action potential of ionic current block in the VL (left) and AL (centre) hiPSC‐CM and hAdultV‐CM (right) models. The four blocker concentrations considered were as follows: 0.1 × IC_50_ = 9%, IC_50_ = 50%, 2 × IC_50_ = 67% and full. For all the models, *I_Kr_* traces report AP after 7 s from block. *I_NaCa_* full block traces refer to 10 beats after block.

**Figure 5:**
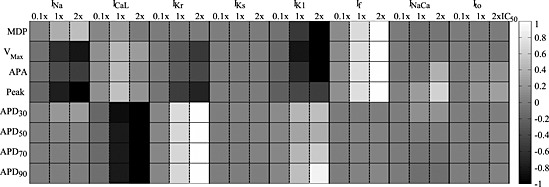
Global comparison of the effect of ionic current blocks on the morphological AP biomarkers simulated by the VL hiPSC‐CM model. Stimulated APs were considered (pacing rate 60 beats min^‐1^). Grey levels represent the % variation of each biomarker (normalized in the interval [−1, 1], black: greatest reduction, white: greatest increment) for each block level. For *I_Kr_*, biomarkers were computed 7 s after block, because for 2 × IC_50_ the AP was prolonged over the next stimulation pulse. APD_xx_, action potential duration at XX% of repolarization.

**Figure 6:**
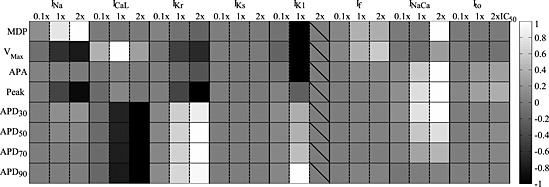
Global comparison of the effect of ionic current blocks on the morphological AP biomarkers simulated by the atrial‐like hiPSC‐CM model. Stimulated APs were considered (pacing rate 80 beats min^‐1^). Grey levels represent the % variation of each biomarker (normalized in the interval [−1, 1], black: greatest reduction, white: greatest increment) for each block level. 2 × IC_50_ was not considered for *I_K1_* due to repolarization failure. For *I_Kr_*, biomarkers were computed 7 s after block, because for 2 × IC_50_ the AP was prolonged over the next stimulation pulse. APD_xx_, action potential duration at XX% of repolarization.

**Figure 7:**
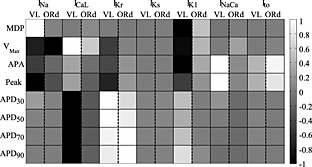
Comparison of the normalized % variation of the biomarkers induced by the blocker concentration 2 × IC_50_ in the VL hiPSC‐CM and the hAdultV‐CM (ORd) models. Biomarkers for *I_Kr_* blocks were computed after 7 s from administration. APD_xx_, action potential duration at XX% of repolarization.

Figures 3 and 4 show the stimulated APs for VL hiPSC‐CM, AL hiPSC‐CM and hAdultV‐CM in control conditions and for the four degrees of block of *I_Na_*, *I_CaL_*, *I_f_*, *I_K1_* and *I_Kr_*, *I_Ks_*, *I_to_ I_NaCa_*. Biomarker values are summarized in Figures 5 and 6 for VL and AL hiPSC‐CM and in Figure S2 for hAdultV‐CM. A specific comparison of the variations induced by 2 × IC_50_ block in VL hiPSC‐CM and hAdultV‐CM APs is presented in Figure 7. Numerical values are presented in Table 2, and in the Supporting Information, Tables S1–S3. Stimulated APs are also compared with spontaneous ones in Tables S4 and S5, showing that the biomarker changes are quite consistent.

#### *I_Na_* *block*

Specific *I_Na_* block is usually obtained by means of tetrodotoxin (Ma *et al*., 2011) or mexiletine (Ma *et al*., 2013). As expected, in all cell types *I_Na_* block affected primarily the AP upstroke, and, therefore, V_Max_ and AP amplitude (Figures 3A, S1 and Tables 2, S1–S5). V_Max_ was strongly reduced by *I_Na_* block in both hiPSC‐CMs (up to −78%). The peak voltage was affected as well, and the VL hiPSC‐CM was found to be more sensitive than the AL hiPSC‐CM.

Similar effects of *I_Na_* block were observed in the hAdultV‐CM upstroke (Figure 3A and Tables 2 and S3), including a dramatic decrease in V_Max_ with the highest levels of block (up to −69%). The peak voltage was slightly less sensitive to *I_Na_* block in simulated hAdultV‐CM than in VL hiPSC‐CM. For full block, the stimulus current had to be increased from 80 to 140 pA·pF^−1^ to trigger an AP due to a significant reduction in excitability.

#### *I_CaL_* *block*

*I_CaL_* has a critical role in sustaining the AP plateau and therefore the APD. In *in vitro* experiments, it is selectively blocked by using nifedipine (Ma *et al*., 2011) or nisoldipine. In our simulations, the *I_CaL_* block‐induced APD shortening was evident in all models, although it was larger in hiPSC‐CMs than in hAdultV‐CM (over 68% vs. less than 30% for full block) (Figure 3B and Tables 2, S1–S5). Of note, due to a marked APD shortening, the spontaneous beating prevailed on pacing at 80 beats·min^−1^ for the AL hiPSC‐CMs, therefore the pacing rate was increased to 100 beats·min^−1^.

We hypothesized that such a different AP shortening in VL hiPSC‐CM and hAdultV‐CM is induced by differences in the *I_NaCa_* between the two models. To test this hypothesis, we replaced the *I_NaCa_* formulation in the hAdultV‐CM model by the one in the VL hiPSC‐CM. The simulations then showed that APD after *I_CaL_* block was close to that obtained in VL hiPSC‐CM (Figure S4, green vs. blue curves). Comparing the time course of *I_NaCa_* during the AP in the original hiPSC‐CM model and in the hAdultV‐CM model with the hiPSC‐CM *I_NaCa_* formulation under *I_CaL_* full block conditions (not shown) shows that the current profiles are similar but the amplitude of the current (both in outward and inward modes) is 3.2 times larger in hiPSC‐CM than in hAdultV‐CM. We then increased the maximal *I_NaCa_* current by 3.2 times and tested the *I_CaL_* full block again, which showed that the *I_NaCa_* increase was almost fully responsible for the APD shortening obtained within the hAdultV‐CM model with the hiPSC‐CM *I_NaCa_* formulation (see in Figure S4 the red curve almost superimposed to the green one). Finally, we also tested the effect of the persistent component of the sodium current *I_NaL_* (not present in the hiPSC‐CM models) in preserving the APD in hAdultV‐CM in conditions of *I_CaL_* block. By fully blocking the *I_NaL_* and *I_CaL_* at the same time in the hAdultV‐CM model with the hiPSC‐CM *I_NaCa_* formulation, an even larger APD_90_ reduction was achieved that almost equalled the shortening shown in hiPSC‐CM after *I_CaL_* block (see in Figure S4 the magenta curve almost superimposed to the blue one). The analysis therefore suggests that the differences in the response of hAdultV‐CM and hiPSC‐CM to *I_CaL_* block are due to differences in *I_NaCa_* and *I_NaL_* currents.

#### *I_Kr_* *and* *I_Ks_* *block*

As shown in Figure 4A and Tables 2, S1–S5, *I_Kr_* block induces a strong APD prolongation for all cell types, at all repolarization phases. A similar APD prolongation was obtained using E4031 (Ma *et al*., 2011) or dofetilide. At the IC_50_ concentration, the APD_90_ increased by about +40% for all the three models. Full *I_Kr_* block induced an even more dramatic prolongation of APD_90,_ +90% and more for both hiPSC‐CMs, and for hAdultV‐CM, early after depolarizations (EADs) were triggered. Note the strong decrease in V_Max_ caused by *I_Kr_* block in hiPSC‐CMs, which was due to the fact that APD prolongation reduces the diastolic interval and therefore limits the *I_Na_* recovery from inactivation and its availability to trigger the next AP. In VL hiPSC‐CM, for 2 × IC_50_ *I_Kr_* block, only 6% of *I_Na_* current was available (computed as product of the inactivation gating variables *h* and *j*) at the end of the diastolic phase. The V_Max_ drop observed in VL hiPSC‐CM was not seen for hAdultV‐CM, in which *I_Na_* had sufficient time to recover from inactivation even after *I_Kr_* block‐induced AP prolongation because, in spite of the similar % prolongation, the absolute APD_90_ value was significantly shorter in hAdultV‐CM than in VL hiPSC‐CM (446 ms vs. 732 ms at 2 × IC_50_).

In Ma *et al*, (2011), 3R4S‐Chromanol 293B was used for *I_Ks_* block but its influence on *I_to_* was also reported (Thomas *et al*., 2003). In Thomas *et al*., (2003) and So *et al*. (2008), HMR‐1556 resulted a more selective blocker of *I_Ks_*, with influence on other currents only at high concentrations. Simulations demonstrated only a minor effect of *I_Ks_* block on the AP shape for both hiPSC‐CMs and hAdultV‐CM (Figure 4B and Tables 2, S1–S5), with APD_90_ shortening <3% for full block. This is also due to the fact that in our *in silico* analysis, the effect of stimulation of β‐adrenoceptors was not considered.

#### *I_K1_* *block*

Simulations show the importance of *I_K1_* in maintaining the MDP in all cell types but with significant quantitative differences (Figure 3D and Tables 2, S1, S2, S4 and S5). *I_K1_* block, which can be performed *in vitro* by means of BaCl_2_ (Sartiani *et al*., 2007), resulted in an elevated MDP (over 9%) in both hiPSC‐CMs, and as a consequence, reduced *I_Na_* availability and a severe reduction in V_Max_ by over 50% for IC_50_ block. The influence of *I_K1_* on APD was also strong for both hiPSC‐CMs, especially for APD_90_, which was prolonged at the IC_50_ by 28% in VL hiPSC‐CM and by 58% in AL hiPSC‐CM. Figure 3D shows the repolarization failure that immediately followed the full *I_K1_* block in both the hiPSC‐CM models (and 2 × IC_50_ block only for AL hiPSC‐CM).

The hAdultV‐CM simulations show a very stable MDP for the three lowest degrees of *I_K1_* block (Figure 3D and Tables 2, S3). In contrast, a complete block induced a marked slowdown of the late repolarization with consequent APD_90_ prolongation, and this was followed by a slight hyperpolarization (+5%) of MDP.

To understand the different behaviours in VL hiPSC‐CM and hAdultV‐CM, we performed additional *in silico* analyses to evaluate the role of *I_Kr_* in compensating for the lack of a repolarizing effect of *I_K1_* in hAdultV‐CM versus VL hiPSC‐CM. As reported in Figure S5A and S5B, the peak *I_Kr_* under AP was half in VL hiPSC‐CM with respect to hAdultV‐CM (0.4 vs. 0.86 pA·pF^−1^) and it was not enough to repolarize the AP in the case of *I_K1_* block. To confirm this result, we simulated, in conditions of full block of *I_K1_*, the effect of doubling *I_Kr_* in hiPSC‐CM (Figure S5C) and halving the *I_Kr_* in hAdultV‐CM (Figure S5D). Figure S5C shows in VL hiPSC‐CM how a doubled *I_Kr_* partly compensates for the absence of *I_K1_*, and restores repolarization. In contrast, Figure S5D illustrates that in hAdultV‐CM, reducing *I_Kr_* enhances the effects of I*_K1_* block, especially during the late repolarization.

#### *I_f_* *block*

*I_f_* can be blocked by means of zatebradine (Sartiani *et al*., 2007) or ivabradine. Figure 3C and S3 show that *I_f_* block has only minor effects on the AP shape in both hiPSC‐CMs, whereas it cannot be simulated in hAdultV‐CM because it lacks *I_f_*. However, in VL hiPSC‐CM, it has some enhancing effects on MDP, V_Max_ and peak voltage (Figure 5 and Tables 2, S1, S2, S4 and S5). As shown in Figure 2, *I_f_* is stronger in VL than in AL hiPSC‐CMs, especially its inward component, which could explain the greater sensitivity of VL hiPSC‐CM to *I_f_* changes. By blocking *I_f_*, the reduced inward component hyperpolarizes MDP up to 3%; especially for VL hiPSC‐CM: this is reflected in an increased V_Max_ and peak voltage.

#### *I_NaCa_* *block*

In Paci *et al*., (2012), nickel was used to block the *I_NaCa_*, but it also affected *I_Kr_* and the T‐type Ca^2+^ current. Recently, the effect of the more specific *I_NaCa_* blocker ORM10103 was tested on canine ventricular cells, but its effects in human ventricular myocytes are still unknown (Kormos *et al*., 2014; Nagy *et al*., 2014). The most important effect caused by *I_NaCa_* block (Figure 4C and Tables 2, S1, S2, S4 and S5) in *in silico* hiPSC‐CMs and in hAdultV‐CM, especially at full block, is the APD_90_ shortening, up to 20% in VL hiPSC‐CM. In VL and AL hiPSC‐CMs, we also observed a lot of additional effects. At full block, MDP was hyperpolarized because of the lack of the inward component of *I_NaCa_*; this hyperpolarization induced a greater activation of *I_Na_* and consequently a greater V_Max_; moreover, as a consequence of the hyperpolarized MDP the *I_NaCa_* block affected the whole AP morphology, with an increased peak voltage and the plateau phase moved towards more positive potentials and a slower repolarization, which made the APD_30_ and APD_50_ longer. Conversely, the phase 3 repolarization was faster and induced APD_90_ shortening. The phase 2 change was even more pronounced in AL hiPSC‐CM, leading to a longer plateau duration, which almost compensated for the shortening obtained during the late repolarization. The full block traces shown in Figure 4C for both the hiPSC‐CM models and the hAdultV‐CM model are illustrative of the *I_NaCa_* block occurring 10 beats after blocker administration: traces at the steady‐state were not considered because of reduced intracellular ion concentrations. Finally, Figures S6 and S7 show the effect of *I_NaCa_* block on the intracellular Na^+^ and Ca^2+^ concentrations.

#### *I_to_* *block*

Results using selective *I_to_* blockers are scarce. In de Haan *et al*. (2006), AVE0118 was reported to be specific for *I_to_* but it also affects the ultrarapid K^+^ current. *I_to_* block resulted in only minor effects in VL and AL hiPSC‐CMs (Figure 4D and Tables 2, S1, S2, S4 and S5), specifically it increased the peak voltage and APD_90_, especially for AL hiPSC‐CM. Also for the hAdultV‐CM (Table S3), *I_to_* block mainly induced an increased peak voltage. *I_to_* block would be expected to induce stronger effects in epicardial hAdultV‐CM with a stronger *I_to_* than the endocardial hAdultV‐CM considered here.

## Discussion

The present study provides a first systematic *in silico* evaluation of the effect of selective ionic current block on the AP shape of VL and AL hiPSC‐CMs, and their comparison to hAdultV‐CM. Quantitative results on AP biomarkers for the three cell types are obtained for four block levels of the eight most important transmembrane cardiac currents. Our main findings are as follows: (i) specific ionic current block induces similar qualitative changes in hiPSC‐CMs and hAdultV‐CM AP biomarkers for most ionic currents; (ii) given the importance of *I_Kr_* block in preclinical drug evaluation, our results highlight that *I_Kr_* block results in nearly identical AP prolongation in VL hiPSC‐CM and hAdultV‐CM for 0.1 × IC_50_ and IC_50,_ and consistent for 2 × IC_50_; (iii) hiPSC‐CMs were more sensitive to *I_CaL_* and *I_K1_* block, compared with hAdultV‐CMs, for example, APD shortening/prolongation was up to more than four times larger after *I_CaL_*/*I_K1_* block; (iv) high levels of *I_K1_* block induced repolarization failure in hiPSC‐CMs, which was not observed in hAdult‐CM (this increased sensitivity can be attributed to a reduced repolarization reserve of hiPSC‐CMs); (v) our mechanistic study revealed the importance of differences in *I_NaCa_*, *I_NaL_* and *I_K1_* between hAdult‐CM and hiPSC‐CMs in modulating their response to ionic current block. Although AL cells are present in *in vitro* preparations, they are a less common phenotype compared with the VL ones, and they are rarely used for drug tests. Therefore, greater attention was placed in this study on the VL hiPSC‐CM model and its comparison to hAdultV‐CM. However, we consider the presentation of the AL hiPSC‐CM simulations useful, especially in the perspective of multicellular aggregates (e.g. multielectrode array and field potential).

By revealing differences and similarities in the sensitivity of hiPSC‐CMs and hAdultV‐CM to ionic current block, our analysis aims to help in the interpretation of further *in vitro* assays. We therefore expect the results of our investigations to support the currently developing synergy between *in vitro* and *in silico* approaches in the field of pharmacology and facilitate further development and use of hiPSC‐CMs as a powerful *in vitro* technology for pharmacological applications in drug development and prescreening, which can be extended to take into consideration patient‐ and pathology‐specificity.

### hiPSC technology

Human‐induced pluripotent stem cell‐derived cardiomyocytes are likely to revolutionize drug testing as their production and use is more convenient and practical than harvesting human adult cells for *in vitro* studies, especially for non‐diseased subjects. Further, they could contribute to the reduction, refinement and replacement of animal experimentation.

Human‐induced pluripotent stem cell‐derived cardiomyocytes have already proved their value as *in vitro* models for investigations into specific pathologies (Moretti *et al*., 2010; Egashira *et al*., 2012; Kujala *et al*., 2012; Terrenoire *et al*., 2013), and due to their patient‐specificity, in identifying drugs for specific diseases (Itzhaki *et al*., 2011, 2012; Matsa *et al*., 2011; Yazawa *et al*., 2011; Jung *et al*., 2012), even though none of them has yet been administered to patients (Knollmann, 2013). Further advances in the development and application of the hiPSC technology for personalized therapies, however, require an in‐depth and systematic evaluation of hiPSC‐CMs and their maturity with a quantitative comparison with hAdultV‐CM, which can be facilitated by *in silico* investigations such as the one presented in this study.

The majority of the most recently published datasets shows not only a high variability in the proposed APs (Table 3), but also immature phenotypes, for example, characterized by spontaneous APs and depolarized MDP. This leads to the still‐open question “how similar are hiPSC‐CMs to adult CMs?” and our study provides the first systematic study to aid in the characterization of similarities and differences.

### Effect of ionic current block on hiPSC‐CMs APs

Selective ionic current block was simulated by reducing the maximum conductance, using a simple pore block model. To investigate dose‐dependency of the effects, four degrees of block were applied for each current. Previous studies (Romero *et al*., 2009, 2011; Pueyo *et al*., 2010; Christophe, 2013; Zemzemi *et al*., 2013) have followed a similar approach to systematically investigate the sensitivity of AP biomarkers to specific ion channel changes. This approach was chosen because (i) selective blocks are important for cardiac drug discovery and (ii) it allows a controlled and systematic comparison between different cell types or models, which is a preliminary step before extending the analysis to more complex drug effects including multichannel actions.

In our study, VL hiPSC‐CMs and hAdultV‐CMs action potentials were simulated using the most comprehensive human models consistent with a large range of human data. Our new results showed a qualitatively similar response to current block between both cell types in several cases: (i) *I_Na_* block induced a reduction of V_Max_, peak voltage and AP amplitude; (ii) *I_CaL_* block shortened the APD; (iii) *I_Kr_* block prolonged the APD; (iv) *I_to_* block increased the peak voltage; and (v) low sensitivity was found to *I_Ks_* block. Nevertheless, quantitative differences were identified, such as (i) *I_CaL_* block shortened APD more in hiPSC‐CMs than in hAdultV‐CM and (ii) *I_K1_* block elevated MDP in hiPSC‐CMs but not in hAdultV‐CM. The mechanisms underlying such similarities and differences were investigated and discussed in the following by using the AP models for specifically designed simulations, which provide a powerful tool to unravel the underlying ionic mechanisms.

### Ionic mechanisms underlying hiPSC‐CM versus hAdultV‐CM similarities and differences

The first main difference we observed between VL hiPSC‐CM and hAdultV‐CM is the stronger APD reduction, induced by *I_CaL_* block, in the first model than in the second one. After testing many hypotheses, for example, the influence of the intracellular Na^+^ and Ca^2+^ concentrations, and removing one by one the currents from the hAdultV‐CM model to assess their contribution in case of *I_CaL_* block, we found that the different extent of APD shortening is mainly due to the different *I_NaCa_* formulations and to the presence in hAdultV‐CM of *I_NaL_*. Concerning *I_NaCa_*, we performed the following *in silico* experiment: we transplanted the hiPSC‐CM formulation of *I_NaCa_* into the hAdultV‐CM model, to evaluate its importance in the observed differences. Of note, this is one of the main advantages of *in silico* modelling, which allows in a relative short time to simulate phenomena and mechanisms too hard or time‐consuming to test *in vitro*. In this sense, the computational analysis offers an interesting mechanistic insight into why the response of the hiPSC‐CMs can be different from that of the adult cells: overexpression of *I_NaCa_* can magnify the effects of *I_CaL_* block. Actually, *I_NaCa_* overexpression in incompletely mature CMs, and thus in hiPSC‐CMs too, is still a matter of debate. Experiments in different animal models show the maximal *I_NaCa_* density being reduced during maturation (Itoh *et al*., 2007). The same trend is also maintained in human cells, where the expression of *I_NaCa_* is higher at the mid‐gestation than at the adult stage (Qu *et al*., 2000). In cardiomyocytes derived from human pluripotent cells, in particular embryonic, it is reported how the *I_NaCa_* expression is larger than in fetal and adult cells (Liu *et al*., 2007; Li *et al*., 2013) and it was confirmed by the current measurements reported in Paci *et al*. (2012), where *I_NaCa_* is shown to be greater at the early developmental stage (15–40 days). However, in Fu *et al*. (2010), *I_NaCa_* was reported to be greater at 90 days than at 40 days post‐differentiation. Our simulation results point out the need for experimental data quantifying this aspect.

The second contribution to the preserved APD in hAdultV‐CM after *I_CaL_* block comes from *I_NaL_*. Indeed, this current is included in the hAdultV‐CM model, whereas it is not in the hiPSC‐CM one, which is mainly based on the data of Ma *et al*. (2011), where no *I_NaL_* was reported. The lack of this influx of positive charges delegates the support of the AP plateau phase to *I_CaL_*, whose block has stronger effects in VL hiPSC‐CM than in hAdultV‐CM. Because *I_NaL_* has been reported in hiPSC‐CMs (Ma *et al*., 2013), in this case, simulation results point out the need for the experimental characterization of this current and its inclusion in the hiPSC‐CMs mathematical models.

The second main difference we observed between hiPSC‐CM and hAdultV‐CM is the extent of the effects of *I_K1_* block, which confirms the critical role played by a non‐completely mature *I_K1_* in stem cells‐derived CMs, in which instability of diastolic potential leads to spontaneous depolarization. Our results show that *I_K1_* block results in significant effects on MDP and APD_90_ in VL hiPSC‐CM, whereas in hAdultV‐CM, MDP remains unaffected and APD_90_ prolongation is modest (<10%). We ascribe the greater sensitivity to *I_K1_* blockade of VL hiPSC‐CM to a reduced reserve of repolarization (Carmeliet, 2006): in hiPSC‐CM, *I_Kr_* is not able to carry out properly those compensatory mechanisms that in hAdultV‐CM allow the reduction of the *I_K1_* block effects.

For cardiotoxicity studies, the greater sensitivity of hiPSC‐CM to drug blocks could be regarded as a useful feature providing clearly detectable indications about how a specific compound affects the cell electrophysiology. However, as shown by our study and *in vitro* experiments (Ma *et al*., 2011; Knollmann, 2013), it is also an important indicator of the fact that the cells the Paci2013 model is based on, and more in general hiPSC‐CMs, were not behaving exactly like adult cells, thus particular care should be used when considering them as fully representative models of adult cells. This oversensitivity is something to be aware of, but not enough to dismiss hiPSC‐CMs as an inadequate model. We know indeed that every *in vivo*, *in vitro* or *in silico* model has advantages and disadvantages in the translation of its results to humans. If anything, this highlights the importance of further studies to achieve better and more effective maturation, characterization and possibly improve (e.g. *I_K1_* overexpression) hiPSC‐CMs. The final goal is an integrated approach, in which compounds are tested in real hiPSC‐CMs, whereas *in silico* simulations with both hiPSC‐CM and hAdultV‐CM will help in interpreting and translating the results to real adult cells *in vivo*. Thus, decisions should be taken from data obtained by running in parallel *in silico* and *in vitro* experiments on hiPSC‐CMs and taking into account both sets of result. It will improve the degree of awareness of hiPSC‐CM descriptive power: if results are consistent, the confidence in them will be higher, otherwise the interpretation of the results should be treated with caution. This scenario also represents a typical field for the application of the new concept of model‐simulation‐experiment (MSE) system (Carusi *et al*., 2012). In this regard models, simulations and experiments are interlaced in a synergic assemblage, whose expressive power for the representation of biological mechanisms progressively grows, as the MSE system itself is constructed.

A further step for cardiotoxicity studies consists in analysing and integrating different levels of details into a multiscale approach. In fact, in order to get an overall understanding of arrhythmogenic mechanisms and how new compounds could affect them, the integration of measurements and simulations at the cellular, tissue, organ and even *in vivo* levels is essential.

Potential applications of our methodology are as follows: (i) *in silico* experiments aimed at evaluating stem cell‐derived CM maturity, for example, by replacing single or multiple ionic currents with their adult versions, in order to compare hiPSC‐CM models with hybrid or partially adult models; (ii) the investigation of the observed high *in vitro* variability by means of *in silico* models derived from patient‐specific and disease‐specific hiPSC‐CMs, for example exploiting specific populations of models (Britton *et al*., 2013; Gemmell *et al*., 2014); and (iii) the analysis of the already known differences between hiPSC‐CMs and hAdultV‐CMs, for example, assessing the impact of a reduced repolarization reserve on *in vitro* models of arrhythmogenic pathologies.

### Future work and limitations

A comparison of published datasets highlights the surprisingly high variability in the APs measured during experiments in hiPSC‐CMs. In the light of such large variations in measurements and cell lines, the construction of *in‐silico* models requires an informed choice on the experimental data to be considered and integrated. In Paci *et al*. (2013a), the hiPSC‐CM models were based on the comprehensive dataset reported in Ma *et al*. (2011), giving only secondary consideration to additional datasets. Of note, this dataset is based on measurements from isolated iCell hiPSC‐CMs by Cellular Dynamics International; therefore, the Paci2013 model reproduces the electrophysiological behaviour of isolated hiPSC‐CMs from this specific commercial line. Other commercial hiPSC‐CMs lines are available (e.g. Cor.4U by Axiogenesis) and many more are produced in single laboratories, thus line‐to‐line variability is an issue. Moreover, hiPSC‐CM biomarkers are dependent on the measurement conditions, for example, isolated cell patch‐clamp versus microelectrode arrays on monolayers. Consequently, a possible limitation of the present study is the general applicability of our results to hiPSC‐CMs obtained in different laboratories and/or from different cell lines, suggesting that specific *in silico* models should be tailored for different hiPSC‐CM lines. However, it is worth noting that the Paci2013 hiPSC‐CM model already comprises the flexibility to reproduce datasets from cell lines different from the one used to develop the model. In Paci *et al*. (2013b, 2014), we showed how, with few changes in the maximal conductances of the ion currents, we reproduced different control APs. Moreover, integrating mutation‐specific data, mutant LQT1 and LQT3 APs were also properly reproduced. These results suggest that the intrinsic behaviour of the cells, and eventually their response to current blockers, could be more conserved than the more commonly measured AP features. Nevertheless, as reported earlier, this aspect needs to be investigated in more depth, for example, by exploiting the populations of models approach.

In this study, we have considered single pore block models, and this approach has proved useful in previous studies (Brennan *et al*., 2009; Zemzemi *et al*., 2013). It includes important information on cardiac sensitivity to drug effects and can be extended to drugs with multichannel actions. In the case of hiPSC‐CMs, however, the situation is even more complex: indeed, for adult cells, studies have published IC_50_ for different drugs, and also for multiple channels (Mirams *et al*., 2011; Davies *et al*., 2012). At present, the same kind of information is not available for hiPSC‐CMs but our studies could be extended once comparable *in vitro* data become available for hiPSC‐CMs. Furthermore, our work focuses on the effect of ionic current block on AP biomarkers rather than EADs and those were observed only for very high degrees of *I_Kr_* block in hAdultV‐CM. In the case of hiPSC‐CMs, the observed high variability reported in the literature also affects the presence of EADs. In particular, in the literature, the occurrence of EADs is reported to be variable (Hoekstra *et al*., 2012) and generally in specific stimulation conditions (e.g. very slow pacing rate such as 30 beats min^‐1^). Moreover, generally not all the cells tested showed the occurrence of EADs, but only a subset of them showed repolarization anomalies (Ma *et al*., 2011). Variations in the occurrence of EADs were observed not only in control cells but also in mutant hiPSC‐CMs derived from patients, for example, in conditions of impaired *I_Kr_* by LQT2 syndrome: in Lahti *et al*. (2012), EADs were observed, but not by Bellin *et al*. (2013). It seems, therefore, that in order to reliably assess the occurrence of this kind of pro‐arrhythmia marker, the intercellular variability should also be taken into account in the computational models. Further studies can extend our hiPSC‐CMs models to investigate mechanisms of EADs generation. Finally, our modelling approach could also be extended to include more complex models of drug/ion channel interactions, specifically when drug‐induced effects on current kinetics need to be evaluated (Di Veroli *et al*., 2013; Romero *et al*., 2014).

## Conclusions

This study presents the first application of hiPSC‐CM *in silico* models for the assessment and comparison of the effects of specific current block on hiPSC‐CM and hAdultV‐CM APs. Qualitatively, the response of all cell types was overall similar but an increased sensitivity was observed with the hiPSC‐CMs, specifically to *I_CaL_* and *I_K1_* block. Quantitatively, we showed how the increased sensitivity of the hiPSC‐CMs was mainly due to the overexpression of *I_NaCa_* in the case of *I_CaL_* block, whereas it was due to the reduced repolarization reserve in the case of *I_K1_* block. Our results are potentially useful for developing synergistic approaches for pharmacological investigations, using combined *in silico* and *in vitro* hiPSC‐CM technologies for safety assessments and target identification during drug development.

## Author contributions

M. P. conducted the experiments, analysed/interpreted data and wrote the article. J. H. designed the experiments, analysed/interpreted data and proofed/revised the article. B. R. designed the experiments, analysed/interpreted data, wrote the article and proofed/revised the article. S. S. designed the experiment, analysed/interpreted data and proofed/revised the article.

## Conflict of interest

None.

## Supporting information


**Table S1a** AP biomarkers changes induced by the different blockade levels in the stimulated ventricular‐like model.
**Table S1b** AP biomarkers percent variations with respect to the control AP induced by the different blockade levels in the stimulated ventricular‐like model.
**Table S2a** AP biomarkers changes induced by the different blockade levels in the stimulated atrial‐like model.
**Table S2b** AP biomarkers percent variations with respect to the control AP induced by the different blockade levels in the stimulated atrial‐like model.
**Table S3a** AP biomarkers changes induced by the different blockade levels in the adult ORd model (I_f_ not present in the ORd model).
**Table S3b** AP biomarkers percent variations with respect to the control AP induced by the different blockade levels in the adult ORd model.
**Table S4** Comparison between spontaneous (gray) and stimulated (white) ventricular‐like hiPSC‐CM APs. Biomarkers are presented as percent variations with respect to the control AP induced by the different blockade levels in the stimulated ventricular‐like model.
**Table S5** Comparison between spontaneous (gray) and stimulated (white) atrial‐like hiPSC‐CM APs. Biomarkers are presented as percent variations with respect to the control AP induced by the different blockade levels in the stimulated atrial‐like model.
**Figure S1** Details of the effect of I_Na_ block on the AP upstroke.
**Figure S2** Global comparison of the effect of ionic current blocks on the morphological action potential (AP) biomarkers simulated by the hAdultV‐CM model. The pacing rate is 60 bpm. Grey levels represent the percent variation of each biomarker (normalized in the interval [−1, 1]) for each block level. MDP: maximum diastolic potential, V_Max_: maximum upstroke velocity, APA: action potential amplitude, Peak: peak voltage, APD_xx_: action potential duration at XX% of repolarization.
**Figure S3** I_f_ block effects on VL hiPSC‐CM and hAL hiPSC‐CM. In panels B and C the effects on MDP and Peak have been detailed, respectively.
**Figure S4** Assessment of the different contributions that sustain the AP in the hAdultV‐CM (ORd) model and comparison with full I_CaL_ block in VL hiMPSC‐CM. Black, green and magenta traces refer to hAdultV‐CM hybridized with the VL hiPSC‐CM I_NaCa_. The red trace refers to the hAdultV‐CM model with its original I_NaCa_ formulation and a 3.2‐fold increase in the maximal current.
**Figure S5** Assessment of the repolarization reserve in VL hiPSC‐CM and hAdultV‐CM. A and B: comparison of different I_K1_ blocks on the APs and the underlying I_Kr_. C: compensation of the I_K1_ block by doubling I_Kr_ (increment to 80 bpm of the pacing rate is needed to avoid spontaneous APs). D: the halved IKr and the consequent slower repolarization show how I_Kr_ compensates the absence of I_K1_ in adult cells.
**Figure S6** Na^+^ concentration in conditions of I_NaCa_ block.
**Figure S7** Ca^2+^ concentration in conditions of I_NaCa_ block. Peak of hAdultV‐CM Ca^2+^ transient equals to 0.022 mM.

Supporting info item
