# Supplementary material for: Human induced pluripotent stem cell‐derived versus adult cardiomyocytes: an in silico electrophysiological study on effects of ionic current block
Source: Br J Pharmacol. 2015 Oct 15;172(21):5147–60. doi: 10.1111/bph.13282 (PMC4629192; doi:10.1111/bph.13282)
Supplement: Supplementary file 1 [file bph0172-5147-sd1.pdf]

Supporting information

Human induced pluripotent stem cell-derived versus adult cardiomyocytes: an *in silico* electrophysiological study on ionic current block effects

Michelangelo Paci, Jari Hyttinen, Blanca Rodriguez, Stefano Severi

Changes to the Paci2013 model

In this paper we used a slightly modified version of the Paci2013 model presented in (Paci et al., 2013).

Inclusion of I_f contribution (I_{fNa}) to the intracellular Na^+ mass balance equation:

$$\frac{dNa_i}{dtime} = -C_m \cdot \frac{I_{Na} + I_{bNa} + 3I_{NaK} + 3I_{NaCa} + I_{fNa}}{F \cdot V_c}$$

$$I_{fNa} = 0.42 \cdot g_f \cdot Xf \cdot (V - E_{Na})$$

Tuning of maximum conductances/currents/fluxes, to balance the intracellular concentrations of Na^+ and Ca^{2+} and the Ca^{2+} concentration in the sarcoplasmic reticulum.

		Ventricular-like	Atrial-like
K_{NaCa}	(A/F)	5978	2989
P_{NaK}	(A/F)	2.2958	1.7678
g_{bNa}	(S/F)	0.95	0.95
g_{bCa}	(S/F)	0.727272	0.727272
$Vmax_{up}$	(mM/s)	---	0.198
C_{rel}	(mM/s)	0	0

Stimulation protocol and simulation of ionic current block

Two different stimulation protocols were used for the VL and AL hiPSC-CM models, due to the different spontaneous beating rate: (i) VL hiPSC-CM was stimulated at 60 bpm with depolarizing current pulses of 5 ms duration and 10 pA/pF amplitude, (ii) AL hiPSC-CM was stimulated at 80 bpm with pulses of 5 ms duration and 9 pA/pF amplitude. The hAdultV-CM was stimulated as in (O'Hara et al., 2011) at 60 bpm with stimulus of 0.5 ms duration and 80 pA/pF amplitude.

For three currents biomarkers were not computed at steady state but in different conditions, in detail:

- 7 s after block for all I_{Kr} blocks;
- 10 beats after block for I_{NaCa} full block;
- immediately after administration of the blocker for I_{K1} full block.

Supplementary tables

Table S1a – AP biomarkers changes induced by the different blockade levels in the stimulated ventricular-like model. [a]: biomarkers computed 7s after I_{Kr} blocker administration. [b]: biomarkers computed after 10 beats after I_{NaCa} full block of, due to non-representative ionic concentration at the steady state.

		MDP (mV)	VMax (V/s)	APA (mV)	Peak (mV)	APD30 (ms)	APD50 (ms)	APD70 (ms)	APD90 (ms)	APD_{ratio}
	Control	-76.2	47.4	115	38.5	258	367	418	469	3.41
I_{Na}	0.1×IC_{50}	-76.2	44.5	113	36.9	267	371	420	470	3.37
	IC_{50}	-76.5	29.0	108	31.7	292	380	421	475	2.82
	2×IC_{50}	-76.7	22.2	106	29.8	296	379	418	473	2.72
	FULL	-76.9	10.2	103	26.3	292	375	416	475	2.40
I_{CaL}	0.1×IC_{50}	-76.2	48.8	115	39.1	225	332	382	431	3.46
	IC_{50}	-76.4	51.6	117	40.4	73	152	191	227	3.18
	2×IC_{50}	-76.1	49.8	115	39.1	47	100	132	162	2.20
	FULL	-75.1	45.7	110	34.9	24	47	71	96	1.05
I_{Kr}	0.1×IC_{50} [a]	-76.1	46.8	114	38.1	272	387	441	493	3.53
	IC_{50} [a]	-76.1	38.0	110	34.1	371	524	584	648	3.86
	2×IC_{50} [a]	-75.9	30.8	108	32.1	420	602	664	732	4.71
	FULL [a]	-71.9	14.1	95	23.6	560	825	884	938	8.26
I_{Ks}	0.1×IC_{50}	-76.2	47.3	115	38.5	259	368	419	470	3.41
	IC_{50}	-76.1	46.9	115	38.4	264	373	425	475	3.48
	2×IC_{50}	-76.1	46.9	114	38.3	266	375	427	477	3.54
	FULL	-76.1	46.7	114	38.2	270	380	432	482	3.60
I_{K1}	0.1×IC_{50}	-75.3	42.1	112	36.5	278	382	435	488	3.05
	IC_{50}	-69.5	22.4	104	34.9	325	435	520	599	1.72
	2×IC_{50}	-64.3	16.8	98	34.1	336	449	561	701	0.91
	FULL	REPOLARIZATION FAILURE								
I_f	0.1×IC_{50}	-76.3	48.6	115	39.0	258	368	420	471	3.52
	IC_{50}	-76.9	54.4	118	41.2	261	376	429	484	3.59
	2×IC_{50}	-77.2	57.4	119	42.1	261	379	432	490	3.60
	FULL	-78.1	64.0	122	44.1	262	386	440	503	3.71
I_{NaCa}	0.1×IC_{50}	-76.0	47.2	115	38.7	260	364	413	460	3.57
	IC_{50}	-75.3	46.3	115	39.4	280	364	402	435	3.73
	2×IC_{50}	-75.7	47.1	117	40.8	286	369	405	433	4.22
	FULL [b]	-84.9	75.2	136	51.4	223	312	342	363	5.77
I_{to}	0.1×IC_{50}	-76.1	47.3	115	38.6	259	368	419	470	3.46
	IC_{50}	-76.1	46.7	115	39.1	263	374	425	476	3.49
	2×IC_{50}	-76.0	46.4	115	39.4	263	376	428	478	3.64
	FULL	-76.0	46.2	116	40.5	263	379	433	482	3.79

Table S1b – AP biomarkers percent variations with respect to the control AP induced by the different blockade levels in the stimulated ventricular-like model. [a]: biomarkers computed 7s after I_{Kr} blocker administration. [b]: biomarkers computed after 10 beats after I_{NaCa} full block of, due to non-representative ionic concentration at the steady state.

		Percent variation (%)								
		MDP	VMax	APA	Peak	APD30	APD50	APD70	APD90	APD_{ratio}
I_{Na}	$0.1\times IC_{50}$	0	-6	-1	-4	3	1	0	0	-1
	IC_{50}	0	-39	-6	-18	13	4	1	1	-17
	$2\times IC_{50}$	1	-53	-7	-23	14	3	0	1	-20
	FULL	1	-78	-10	-32	13	2	-1	1	-30
I_{CaL}	$0.1\times IC_{50}$	0	3	1	1	-13	-10	-9	-8	1
	IC_{50}	0	9	2	5	-72	-59	-54	-52	-7
	$2\times IC_{50}$	0	5	0	2	-82	-73	-68	-66	-35
	FULL	-1	-4	-4	-9	-91	-87	-83	-80	-69
I_{Kr}	$0.1\times IC_{50}$ [a]	0	-1	0	-1	5	5	5	5	4
	IC_{50} [a]	0	-20	-4	-12	44	43	40	38	13
	$2\times IC_{50}$ [a]	0	-35	-6	-17	63	64	59	56	38
	FULL [a]	-6	-70	-17	-39	117	125	111	100	142
I_{Ks}	$0.1\times IC_{50}$	0	0	0	0	0	0	0	0	0
	IC_{50}	0	-1	0	0	2	2	2	1	2
	$2\times IC_{50}$	0	-1	0	-1	3	2	2	2	4
	FULL	0	-1	0	-1	4	4	3	3	6
I_{K1}	$0.1\times IC_{50}$	-1	-11	-3	-5	8	4	4	4	-11
	IC_{50}	-9	-53	-9	-10	26	18	24	28	-49
	$2\times IC_{50}$	-16	-65	-14	-12	30	22	34	50	-73
	FULL	REPOLARIZATION FAILURE								
I_f	$0.1\times IC_{50}$	0	3	1	1	0	0	0	1	3
	IC_{50}	1	15	3	7	1	2	3	3	5
	$2\times IC_{50}$	1	21	4	9	1	3	3	4	6
	FULL	3	35	7	15	2	5	5	7	9
I_{NaCa}	$0.1\times IC_{50}$	0	0	0	0	1	-1	-1	-2	5
	IC_{50}	-1	-2	0	2	8	-1	-4	-7	9
	$2\times IC_{50}$	-1	-1	2	6	11	1	-3	-8	24
	FULL [b]	11	59	19	33	-14	-15	-18	-22	69
I_{to}	$0.1\times IC_{50}$	0	0	0	0	0	0	0	0	2
	IC_{50}	0	-1	0	1	2	2	2	1	2
	$2\times IC_{50}$	0	-2	1	2	2	2	2	2	7
	FULL	0	-3	2	5	2	3	4	3	11

Table S2a – AP biomarkers changes induced by the different blockade levels in the stimulated atrial-like model. [a]: biomarkers computed 7s after I_{Kr} blocker administration. [b]: biomarkers computed after 10 beats after I_{NaCa} full block of, due to non-representative ionic concentration at the steady state.

		MDP (mV)	VMax (V/s)	APA (mV)	Peak (mV)	APD30 (ms)	APD50 (ms)	APD70 (ms)	APD90 (ms)	APD_{ratio}
	Control	-71.3	33.9	100	28.8	167	222	267	357	1.09
I_{Na}	$0.1 \times IC_{50}$	-71.5	31.9	101	29.1	169	223	268	358	1.11
	IC_{50}	-72.6	21.6	100	27.8	177	229	270	361	1.11
	$2 \times IC_{50}$	-72.9	17.2	100	26.7	178	230	269	358	1.15
	FULL	-73.6	10.2	99	25.3	176	238	279	366	1.24
I_{CaL}	$0.1 \times IC_{50}$	-71.2	35.5	100	28.6	155	206	247	323	1.19
	IC_{50}	-71.2	37.9	101	29.6	91	133	163	198	1.62
	$2 \times IC_{50}$	-71.2	35.0	100	28.7	65	102	131	159	1.59
	FULL	-71.1	30.5	92	21.1	31	58	86	114	1.05
I_{Kr}	$0.1 \times IC_{50}$ [a]	-71.4	32.8	100	29.1	173	230	281	378	1.08
	IC_{50} [a]	-70.1	23.9	98	27.8	209	294	379	499	1.03
	$2 \times IC_{50}$ [a]	-68.6	19.8	95	26.6	227	333	438	557	1.10
	FULL [a]	-60.3	11.7	83	22.3	257	424	580	678	1.47
I_{Ks}	$0.1 \times IC_{50}$	-71.3	34.0	100	28.9	167	222	267	357	1.11
	IC_{50}	-71.3	33.7	100	28.8	168	223	269	359	1.12
	$2 \times IC_{50}$	-71.3	33.7	100	28.8	169	224	270	359	1.10
	FULL	-71.2	33.6	100	28.8	170	225	271	361	1.10
I_{K1}	$0.1 \times IC_{50}$	-70.2	29.9	100	29.4	174	231	281	376	1.03
	IC_{50}	-57.0	13.2	85	28.3	192	264	349	563	0.44
	$2 \times IC_{50}$	REPOLARIZATION FAILURE								
	FULL									
I_f	$0.1 \times IC_{50}$	-71.4	34.2	100	28.9	168	223	268	358	1.10
	IC_{50}	-71.9	35.4	101	29.3	170	226	272	366	1.09
	$2 \times IC_{50}$	-71.9	36.1	101	29.6	170	226	272	365	1.09
	FULL	-72.9	37.4	103	30.1	174	231	276	378	1.04
I_{NaCa}	$0.1 \times IC_{50}$	-71.0	33.2	101	30.3	176	231	274	349	1.31
	IC_{50}	-70.9	32.3	107	35.9	220	282	319	357	2.25
	$2 \times IC_{50}$	-72.9	34.9	111	38.5	236	301	337	369	2.74
	FULL [b]	-85.6	10.5	141	55.6	171	255	290	315	4.57
I_{to}	$0.1 \times IC_{50}$	-71.2	33.7	101	29.6	168	223	269	358	1.12
	IC_{50}	-71.0	32.5	103	31.5	171	231	279	366	1.23
	$2 \times IC_{50}$	-70.9	32.0	103	32.4	172	234	283	369	1.27
	FULL	-70.7	31.1	105	34.2	174	241	292	375	1.38

Table S2b – AP biomarkers percent variations with respect to the control AP induced by the different blockade levels in the stimulated atrial-like model. [a]: biomarkers computed 7s after I_{Kr} blocker administration. [b]: biomarkers computed after 10 beats after I_{NaCa} full block of, due to non-representative ionic concentration at the steady state.

		Percent variation (%)								
		MDP	VMax	APA	Peak	APD30	APD50	APD70	APD90	APD_{ratio}
I_{Na}	$0.1\times IC_{50}$	0	-6	1	1	1	1	0	0	2
	IC_{50}	2	-36	0	-4	6	3	1	1	1
	$2\times IC_{50}$	2	-49	0	-7	7	4	1	0	6
	FULL	3	-70	-1	-12	5	7	4	3	14
I_{CaL}	$0.1\times IC_{50}$	0	5	0	-1	-7	-7	-7	-9	9
	IC_{50}	0	12	1	3	-45	-40	-39	-44	49
	$2\times IC_{50}$	0	3	0	0	-61	-54	-51	-55	46
	FULL	0	-10	-8	-27	-82	-74	-68	-68	-3
I_{Kr}	$0.1\times IC_{50}$ [a]	0	-3	0	1	3	4	5	6	-1
	IC_{50} [a]	-2	-30	-2	-4	25	33	42	40	-5
	$2\times IC_{50}$ [a]	-4	-42	-5	-8	36	50	64	56	1
	FULL [a]	-15	-65	-17	-23	54	92	117	90	35
I_{Ks}	$0.1\times IC_{50}$	0	0	0	0	0	0	0	0	1
	IC_{50}	0	-1	0	0	1	1	1	1	3
	$2\times IC_{50}$	0	-1	0	0	1	1	1	1	1
	FULL	0	-1	0	0	2	2	2	1	1
I_{K1}	$0.1\times IC_{50}$	-2	-12	-1	2	4	4	5	5	-6
	IC_{50}	-20	-61	-15	-2	15	19	31	58	-59
	$2\times IC_{50}$	REPOLARIZATION FAILURE								
	FULL									
I_f	$0.1\times IC_{50}$	0	1	0	0	0	0	0	0	1
	IC_{50}	1	4	1	2	2	2	2	3	0
	$2\times IC_{50}$	1	7	1	3	2	2	2	2	0
	FULL	2	10	3	4	4	4	3	6	-4
I_{NaCa}	$0.1\times IC_{50}$	0	-2	1	5	5	4	3	-2	20
	IC_{50}	-1	-5	7	24	32	27	19	0	106
	$2\times IC_{50}$	2	3	11	33	41	36	26	3	151
	FULL [b]	20	211	41	93	2	15	8	-12	319
I_{to}	$0.1\times IC_{50}$	0	-1	1	2	1	1	1	0	3
	IC_{50}	0	-4	2	9	2	4	4	3	13
	$2\times IC_{50}$	-1	-6	3	12	3	6	6	4	16
	FULL	-1	-8	5	19	4	9	9	5	26

Table S3a – AP biomarkers changes induced by the different blockade levels in the adult ORd model (I_f not present in the ORd model). [a]: biomarkers computed 7s after I_{Kr} blocker administration. [b]: biomarkers computed after 10 beats after I_{NaCa} full block

		MDP (mV)	VMax (V/s)	APA (mV)	Peak (mV)	APD30 (ms)	APD50 (ms)	APD70 (ms)	APD90 (ms)	APD_{ratio}
	Control	-88.0	259	128	40.0	166	208	240	268	1.60
I_{Na}	0.1×IC_{50}	-88.0	237	127	38.9	168	208	242	269	1.59
	IC_{50}	-88.0	125	125	36.8	173	212	245	273	1.63
	2×IC_{50}	-88.0	80	125	36.7	171	211	244	272	1.60
	FULL	-88.2	80	40	-48.0	62	77	89	100	1.81
I_{CaL}	0.1×IC_{50}	-88.1	260	128	39.9	162	204	237	263	1.83
	IC_{50}	-88.1	263	126	38.4	139	178	209	237	1.50
	2×IC_{50}	-88.1	266	126	37.9	126	165	199	225	1.52
	FULL	-88.1	273	126	37.5	82	131	172	200	1.73
I_{Kr}	0.1×IC_{50} [a]	-87.9	258	128	40.0	175	218	254	282	1.56
	IC_{50} [a]	-87.9	260	128	40.5	215	282	339	379	1.76
	2×IC_{50} [a]	-87.9	259	128	40.5	239	324	401	446	1.64
	FULL [a]	REPOLARIZATION FAILURE								
I_{Ks}	0.1×IC_{50}	-88.0	259	128	40.0	166	209	240	269	1.53
	IC_{50}	-88.0	259	128	40.1	170	215	249	276	1.82
	2×IC_{50}	-88.0	259	128	40.1	174	217	250	279	1.53
	FULL	-87.9	259	128	39.8	180	223	258	285	1.89
I_{K1}	0.1×IC_{50}	-88.0	259	128	40.1	165	208	241	269	1.72
	IC_{50}	-88.2	262	128	40.2	166	208	242	280	1.41
	2×IC_{50}	-88.3	264	129	40.4	166	208	242	287	1.16
	FULL	-92.8	273	136	42.8	165	208	248	352	0.94
I_{NaCa}	0.1×IC_{50}	-88.0	259	128	40.1	163	205	238	265	1.67
	IC_{50}	-88.0	261	128	40.2	150	190	221	249	1.47
	2×IC_{50}	-88.0	262	128	40.2	149	185	216	244	1.31
	FULL[b]	-88.0	262	128	40.2	142	176	204	229	1.46
I_{to}	0.1×IC_{50}	-88.0	259	128	40.3	166	207	240	267	1.61
	IC_{50}	-88.0	259	129	41.4	164	207	240	268	1.76
	2×IC_{50}	-88.0	259	130	41.9	162	205	240	268	1.87
	FULL	-87.9	259	131	43.1	160	205	239	267	1.95

Table S3b – AP biomarkers percent variations with respect to the control AP induced by the different blockade levels in the adult ORd model. [a]: biomarkers computed 7s after I_{Kr} blocker administration. [b]: biomarkers computed after 10 beats after I_{NaCa} full block

		Percent variation (%)								
		MDP	VMax	APA	Peak	APD30	APD50	APD70	APD90	APD_{ratio}
I_{Na}	0.1×IC_{50}	0	-8	-1	-3	2	0	1	0	-1
	IC_{50}	0	-52	-3	-8	4	2	2	2	2
	2×IC_{50}	0	-69	-3	-8	3	2	2	1	0
	FULL	0	-46	-3	-11	-1	2	2	1	13
I_{CaL}	0.1×IC_{50}	0	1	0	0	-2	-2	-1	-2	14
	IC_{50}	0	2	-1	-4	-16	-14	-13	-12	-6
	2×IC_{50}	0	3	-2	-5	-24	-21	-17	-16	-5
	FULL	0	5	-2	-6	-50	-37	-28	-25	8
I_{Kr}	0.1×IC_{50} [a]	0	0	0	0	6	5	6	5	-3
	IC_{50} [a]	0	0	0	1	30	36	41	41	10
	2×IC_{50} [a]	0	0	0	1	45	56	67	66	2
	FULL [a]	REPOLARIZATION FAILURE								
I_{Ks}	0.1×IC_{50}	0	0	0	0	0	1	0	0	-5
	IC_{50}	0	0	0	0	3	4	4	3	14
	2×IC_{50}	0	0	0	0	5	4	4	4	-5
	FULL	0	0	0	-1	9	7	8	6	18
I_{K1}	0.1×IC_{50}	0	0	0	0	-1	0	1	0	8
	IC_{50}	0	1	0	1	0	0	1	4	-12
	2×IC_{50}	0	2	1	1	0	0	1	7	-28
	FULL	5	5	6	7	0	0	3	31	-41
I_{NaCa}	0.1×IC_{50}	0	0	0	0	-1	-1	-1	-1	4
	IC_{50}	0	1	0	0	-9	-8	-8	-7	-8
	2×IC_{50}	0	1	0	1	-10	-11	-10	-9	-18
	FULL [b]	0	1	0	0	-15	-15	-15	-15	-9
I_{to}	0.1×IC_{50}	0	0	0	1	0	0	0	0	1
	IC_{50}	0	0	1	3	-1	-1	0	0	10
	2×IC_{50}	0	0	2	5	-2	-1	0	0	17
	FULL	0	0	2	8	-4	-1	0	-1	22

Table S4– Comparison between spontaneous (GRAY) and stimulated (WHITE) ventricular-like hiPSC-CM APs. Biomarkers are presented as percent variations with respect to the control AP induced by the different blockade levels in the stimulated ventricular-like model. [a]: biomarkers computed 7s after I_{Kr} blocker administration. [b]: biomarkers computed after 10 beats after I_{NaCa} full block.

		Percent variation (%)							
		MDP	VMax	APA	Peak	APD30	APD50	APD70	APD90
I_{Na}	$0.1{\times}IC_{50}$	0	-13	-1	-6	2	2	1	0
		0	-6	-1	-4	3	3	1	0
	IC_{50}	NO SPONTANEOUS APs							
		0	-39	-6	-18	13	13	4	1
	$2{\times}IC_{50}$	NO SPONTANEOUS APs							
		1	-53	-7	-23	14	3	0	1
I_{CaL}	$0.1{\times}IC_{50}$	0	-2	-2	-6	-14	-12	-11	-9
		0	3	1	1	-13	-10	-9	-8
	IC_{50}	-2	-17	-11	-36	-67	-56	-50	-44
		0	9	2	5	-72	-59	-54	-52
	$2{\times}IC_{50}$	-3	-17	-14	-46	-79	-71	-65	-58
		0	5	0	2	-82	-73	-68	-65
I_{Kr}	$0.1{\times}IC_{50}$ [a]	0	1	0	1	4	5	4	4
		0	-1	0	-1	5	5	5	5
	IC_{50} [a]	0	3	1	2	31	34	32	30
		0	-20	-4	-12	44	43	40	38
	$2{\times}IC_{50}$ [a]	0	3	1	2	47	52	50	45
		0	-35	-6	-17	63	64	59	56
I_{Ks}	$0.1{\times}IC_{50}$	0	0	0	0	0	0	0	0
		0	0	0	0	0	0	0	0
	IC_{50}	0	0	0	0	1	1	1	1
		0	-1	0	0	2	2	2	1
	$2{\times}IC_{50}$	0	0	0	0	2	2	1	1
		0	-1	0	-1	2	2	2	2
I_{K1}	$0.1{\times}IC_{50}$	-1	-18	-1	-2	8	5	5	6
		-1	-11	-3	-5	8	4	4	4
	IC_{50}	-9	-79	-6	2	39	31	36	48
		-9	-53	-9	-9	26	18	24	28
	$2{\times}IC_{50}$	-15	-80	-9	7	47	39	54	78
		-16	-65	-14	-12	30	22	34	50
I_f	$0.1{\times}IC_{50}$	0	9	1	4	0	1	1	1
		0	3	1	1	0	0	0	1
	IC_{50}	1	58	7	24	-3	3	5	5
		1	15	3	7	1	2	3	3
	$2{\times}IC_{50}$	2	88	10	35	-5	5	7	6
		1	21	4	9	1	3	3	4
I_{NaCa}	$0.1{\times}IC_{50}$	0	1	0	2	0	-1	-2	-2
		0	0	0	0	1	-1	-1	-2
	IC_{50}	-1	5	4	16	6	-2	-6	-8
		-1	-2	0	2	8	-1	-4	-7
	$2{\times}IC_{50}$	-1	6	6	25	10	0	-5	-8
		-1	-1	2	6	11	1	-3	-8
I_{to}	$0.1{\times}IC_{50}$	0	0	0	0	0	0	0	0
		0	0	0	0	0	0	0	0
	IC_{50}	0	0	1	3	0	1	1	1
		0	-1	0	1	2	2	2	1
	$2{\times}IC_{50}$	0	0	1	5	0	1	2	2
		0	-2	1	2	2	2	2	2

Table S5 – Comparison between spontaneous (GRAY) and stimulated (WHITE) atrial-like hiPSC-CM APs. Biomarkers are presented as percent variations with respect to the control AP induced by the different blockade levels in the stimulated atrial-like model. [a]: biomarkers computed 7s after I_{Kr} blocker administration. [b]: biomarkers computed after 10 beats after I_{NaCa} full block.

		Percent variation (%)							
		MDP	VMax	APA	Peak	APD30	APD50	APD70	APD90
I_{Na}	0.1×IC_{50}	0	-9	0	0	2	2	1	0
		0	-6	1	1	1	1	0	0
	IC_{50}	1	-59	0	-5	9	9	7	4
		2	-36	0	-4	6	3	1	1
	2×IC_{50}	2	-71	0	-4	23	25	27	16
		2	-49	0	-7	6	4	1	0
I_{CaL}	0.1×IC_{50}	0	-3	-2	-6	-9	-9	-10	-11
		0	5	0	-1	-7	-7	-7	-9
	IC_{50}	-2	-29	-13	-43	-41	-41	-41	-41
		0	12	1	3	-45	-40	-39	-44
	2×IC_{50}	-3	-42	-20	-67	-53	-51	-49	-48
		0	3	0	0	-61	-54	-51	-55
I_{Kr}	0.1×IC_{50} [a]	0	-3	0	2	7	8	9	8
		0	-3	0	1	3	4	5	6
	IC_{50} [a]	-2	-23	3	16	65	73	81	70
		-2	-30	-2	-4	25	33	42	40
	2×IC_{50} [a]	-4	-36	4	26	106	123	140	116
		-4	-42	-5	-8	36	50	64	56
I_{Ks}	0.1×IC_{50}	0	0	0	0	0	0	0	0
		0	0	0	0	0	0	0	0
	IC_{50}	0	-1	0	0	1	1	1	1
		0	-1	0	0	1	1	1	1
	2×IC_{50}	0	-1	0	0	1	1	1	1
		0	-1	0	0	1	1	1	1
I_{K1}	0.1×IC_{50}	-1	-21	-1	0	5	5	6	8
		-2	-12	-1	2	4	4	5	5
	IC_{50}	-14	-84	-11	-5	21	25	45	76
		-20	-61	-15	-2	15	19	31	58
	2×IC_{50}	REPOLARIZATION FAILURE							
		REPOLARIZATION FAILURE							
I_f	0.1×IC_{50}	0	4	0	1	1	1	1	0
		0	1	0	0	0	0	0	0
	IC_{50}	1	30	3	7	4	5	5	3
		1	4	1	2	2	2	2	3
	2×IC_{50}	1	44	4	12	5	7	6	4
		1	7	1	2	2	2	2	2
I_{NaCa}	0.1×IC_{50}	0	-3	1	3	5	4	2	-2
		0	-2	1	5	5	4	3	-2
	IC_{50}	-1	-21	6	23	36	29	20	3
		-1	-5	7	24	32	27	19	0
	2×IC_{50}	1	-26	9	32	51	42	30	9
		2	3	11	33	41	36	26	3
I_{to}	0.1×IC_{50}	0	-1	0	1	0	1	1	0
		0	-1	1	2	1	1	1	0
	IC_{50}	0	-3	2	7	1	3	3	2
		0	-4	2	9	2	4	4	3
	2×IC_{50}	0	-4	2	9	1	4	4	3
		-1	-6	3	12	3	6	6	4

Supplementary figures

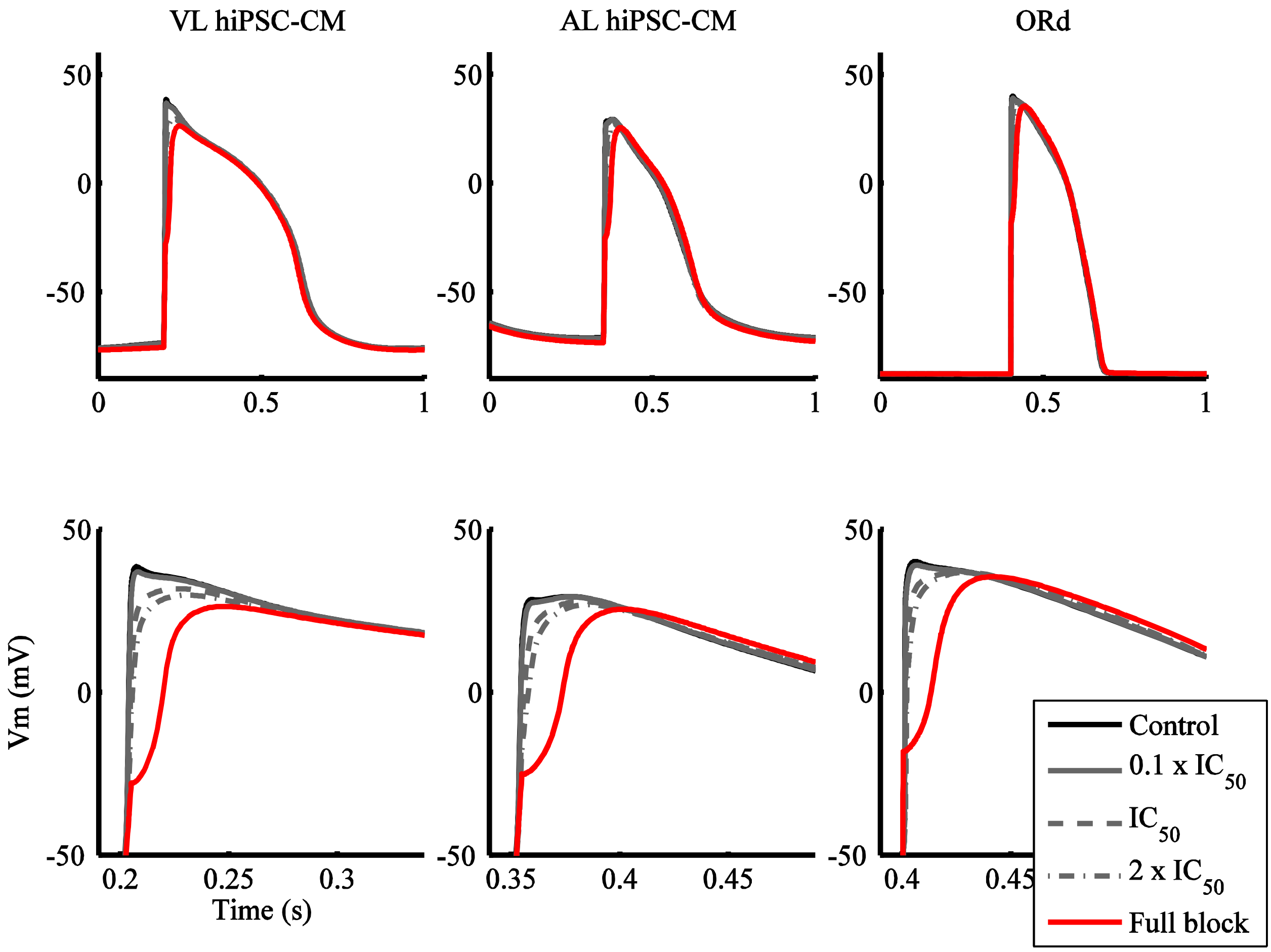

Figure S1 – Details of the effect of I_{Na} block on the upstroke.

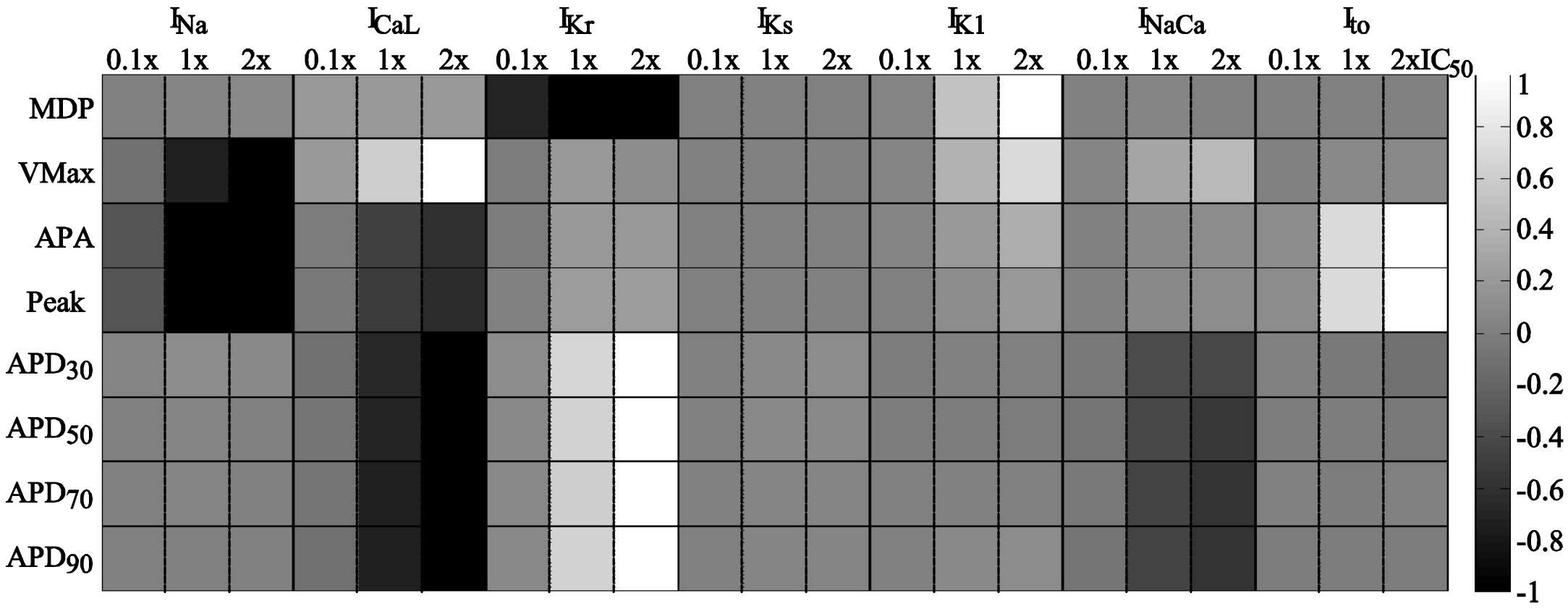

Figure S2 - Global comparison of the effect of ionic current blocks on the morphological action potential (AP) biomarkers simulated by the hAdultV-CM model. The pacing rate is 60 bpm. Grey levels represent the percent variation of each biomarker (normalized in the interval [-1, 1]) for each block level. MDP: maximum diastolic potential, V_{Max}: maximum upstroke velocity, APA: action potential amplitude, Peak: peak voltage, APD_{xx}: action potential duration at XX% of repolarization.

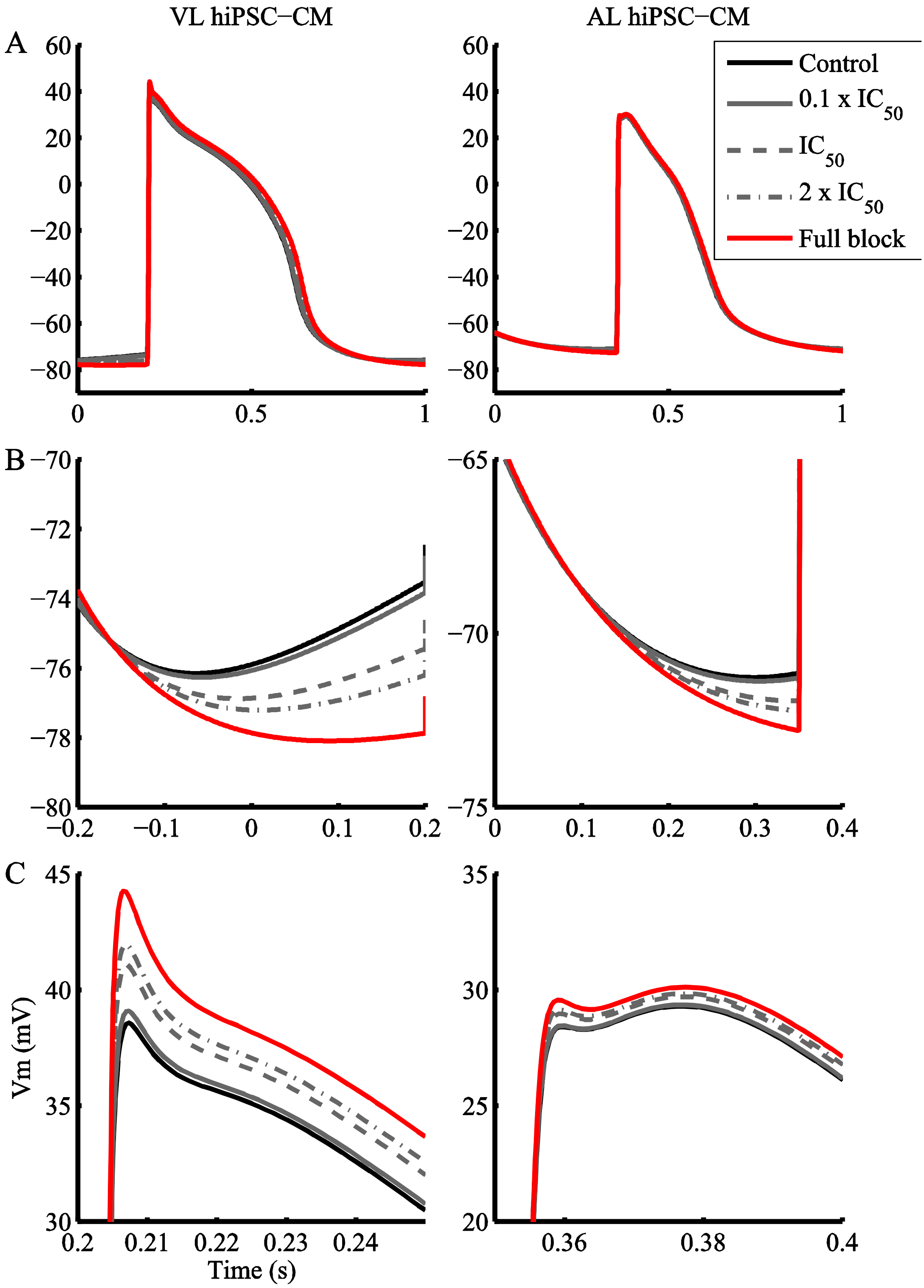

Figure S3 – I_f block effects on VL hiPSC-CM and hAL hiPSC-CM. In panels B and C the effects on MDP and Peak have been detailed, respectively.

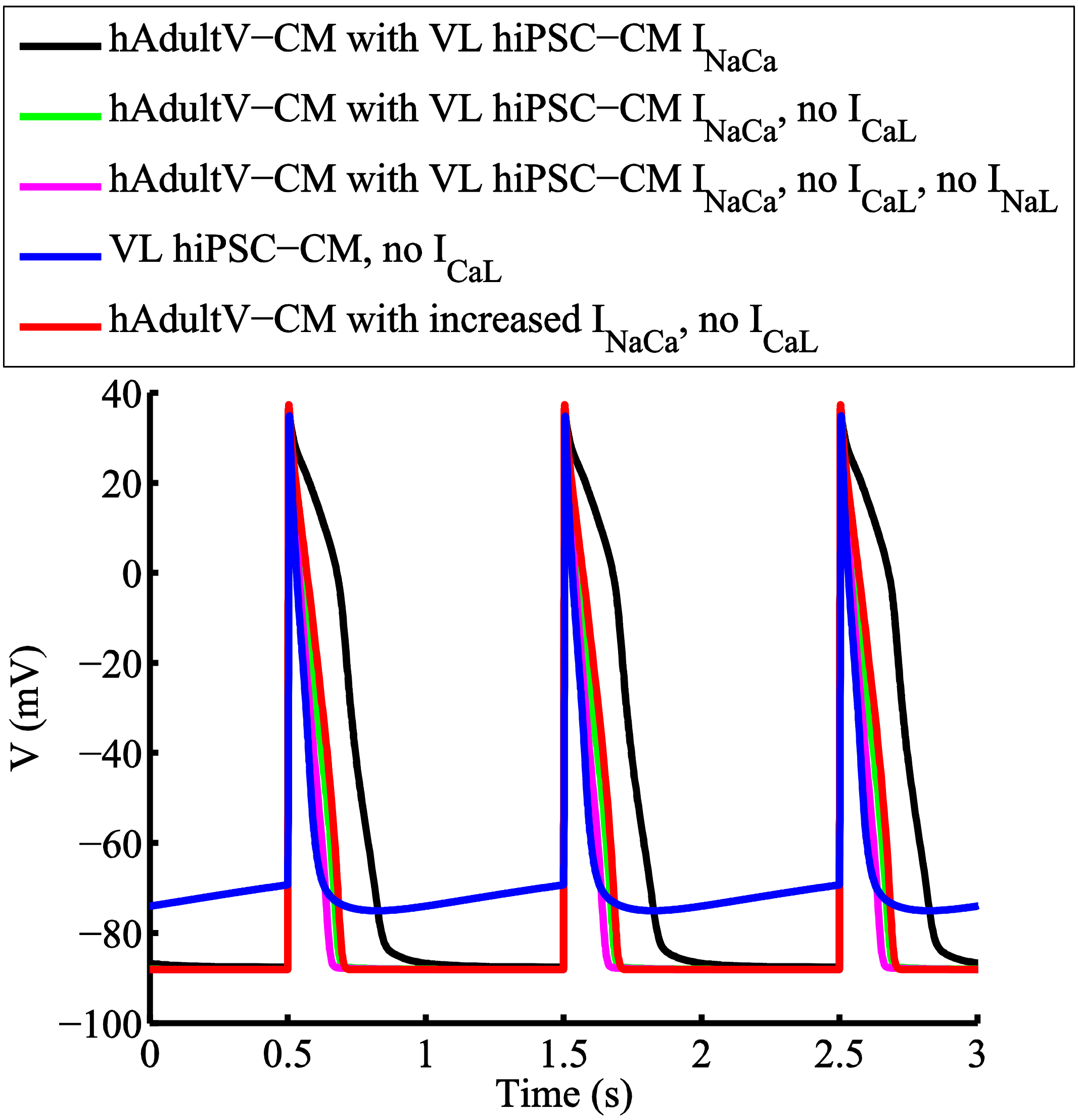

Figure S4 – Assessment of the different contributions that sustain the AP In the hAdultV-CM (ORd) model and comparison with full I_{CaL} block in VL hiMPSC-CM. Black, green and magenta traces refer to hAdultV-CM hybridized with the VL hiPSC-CM I_{NaCa}. The red trace refers to the hAdultV-CM model where its original I_{NaCa}

was increased 3.2 times.

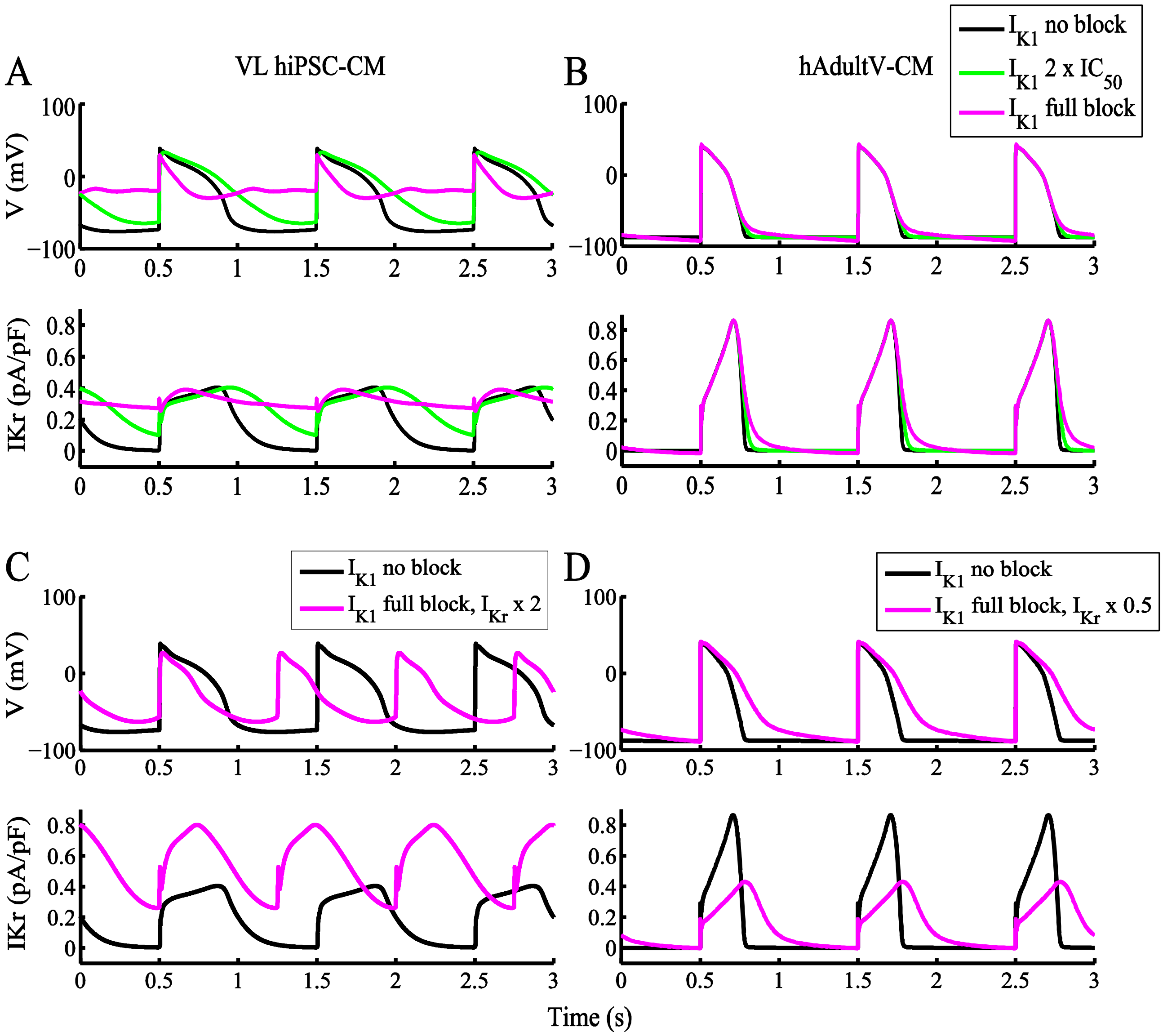

Figure S5 – Assessment of the repolarization reserve in VL hiPSC-CM and hAdultV-CM. A and B: comparison of different I_{K1} blocks on the APs and the underlying I_{Kr}. C: compensation of the I_{K1} block by doubling I_{Kr} (increment to 80 bpm of the pacing rate is needed to avoid spontaneous APs). D: the halved I_{Kr} and the consequent slower repolarization show how I_{Kr} compensates the absence of I_{K1} in adult cells.

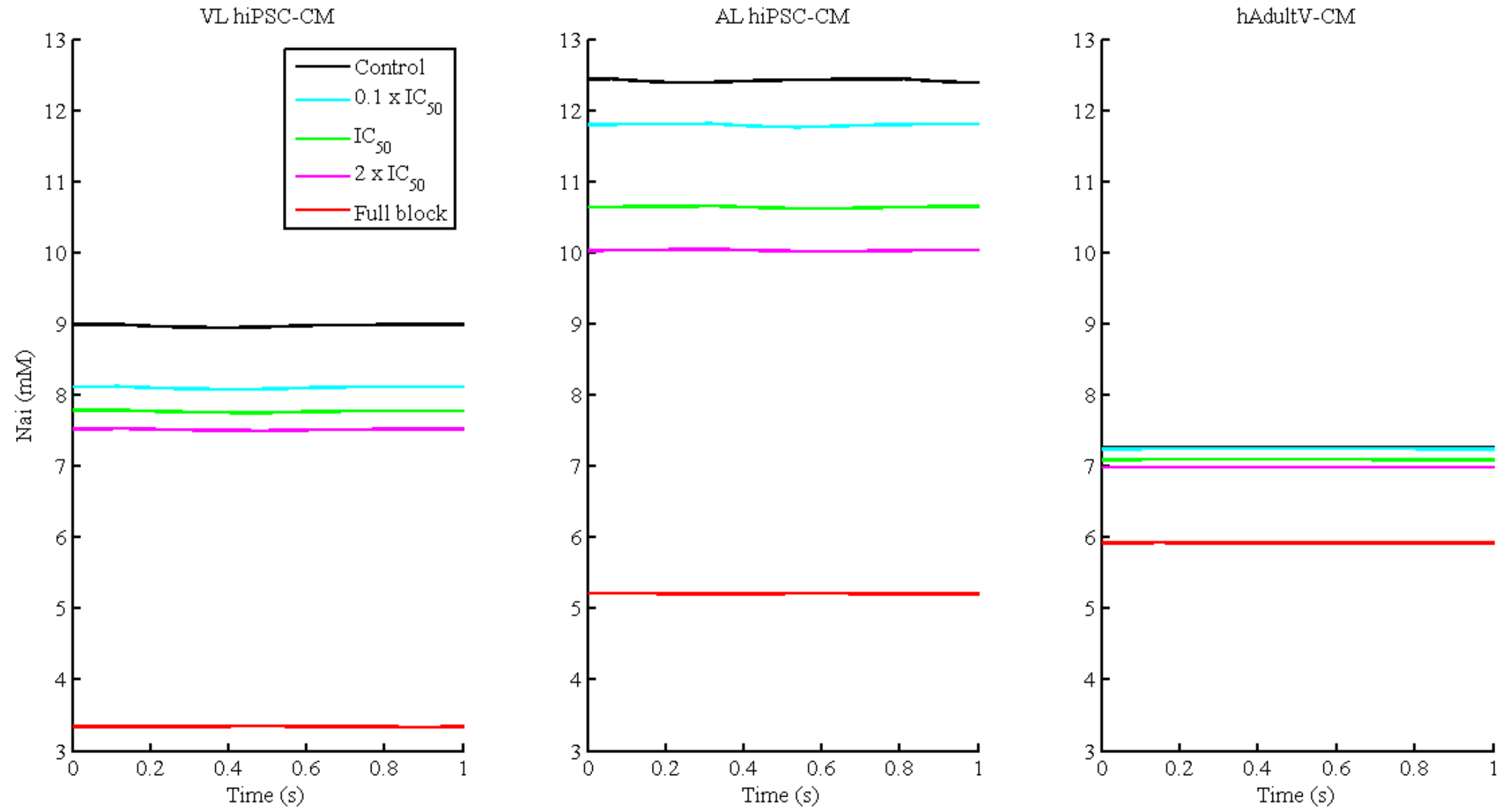

Figure S6 – Na^+ concentration in conditions of I_{NaCa} block.

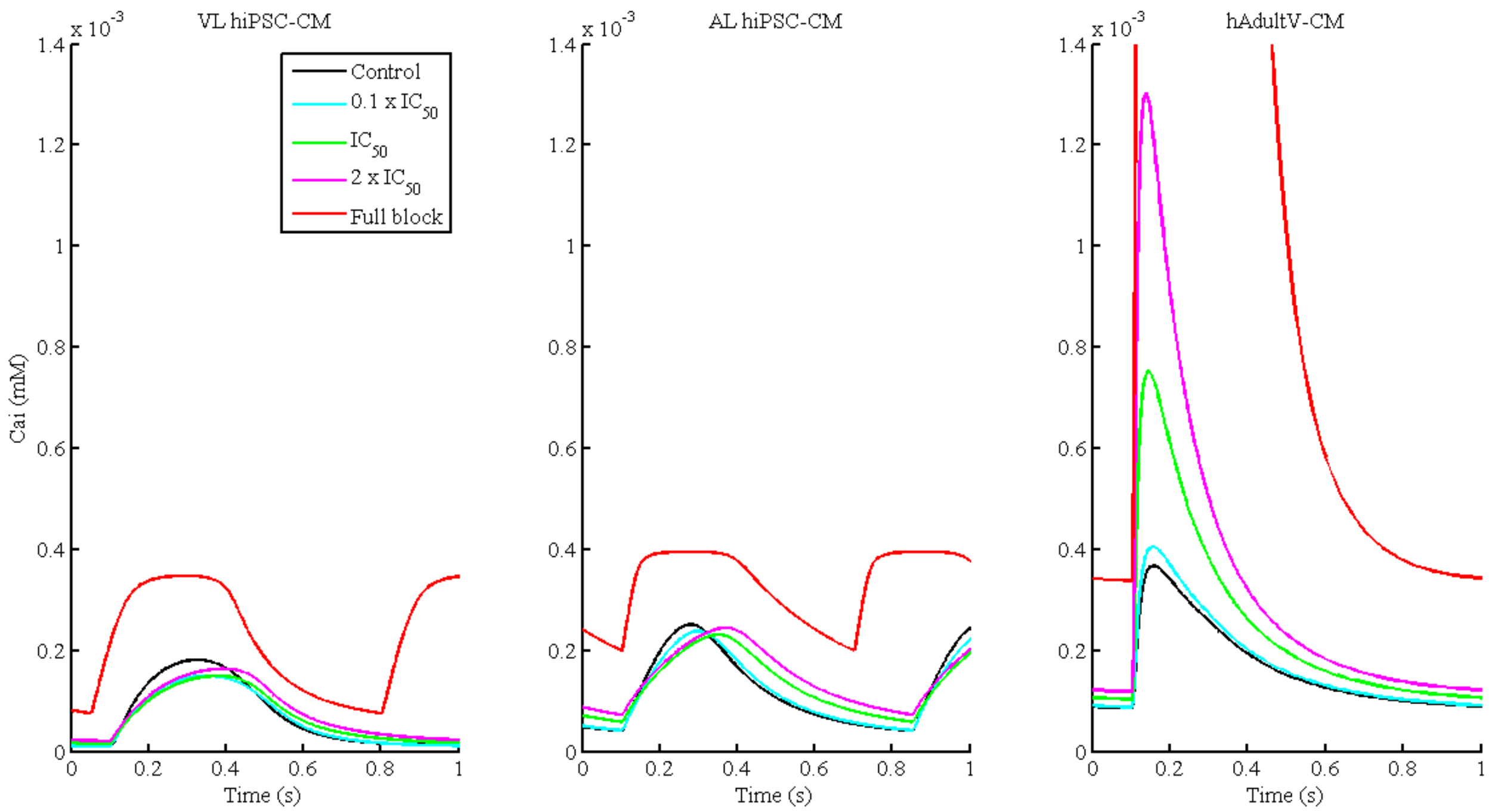

Figure S7 – Ca^{2+} concentration in conditions of I_{NaCa} block. Peak of hAdultV-CM Ca^{2+} transient equals to 0.022 mM.

Bibliography

O'Hara T, Virág L, Varró A, and Rudy Y (2011). Simulation of the Undiseased Human Cardiac Ventricular Action Potential: Model Formulation and Experimental Validation. PLoS Comput Biol 7: e1002061.

Paci M, Hyttinen J, Aalto-Setälä K, and Severi S (2013). Computational models of ventricular- and atrial-like human induced pluripotent stem cell derived cardiomyocytes. Ann Biomed Eng 41: 2334–2348.
